# Structural landscape of the degrading 26S proteasome reveals conformation-specific binding of TXNL1

**DOI:** 10.1038/s41594-025-01695-2

**Published:** 2025-11-06

**Authors:** Connor Arkinson, Christine L. Gee, Zeyuan Zhang, Ken C. Dong, Andreas Martin

**Affiliations:** 1 California Institute for Quantitative Biosciences, University of California at Berkeley, Berkeley, CA USA; 2 Department of Molecular and Cell Biology, University of California at Berkeley, Berkeley, CA USA; 3 Howard Hughes Medical Institute, University of California at Berkeley, Berkeley, CA USA; 4 Biophysics Graduate Program, University of California at Berkeley, Berkeley, CA USA

**Keywords:** Proteases, Cryoelectron microscopy, Enzyme mechanisms, Proteasome, Proteasome

## Abstract

The 26S proteasome targets many cellular proteins for degradation during homeostasis and quality control. Proteasome-interacting cofactors modulate these functions and aid in substrate degradation. Here we solve high-resolution structures of the redox active cofactor TXNL1 bound to the human 26S proteasome at saturating and substoichiometric concentrations by time-resolved cryo-electron microscopy (cryo-EM). We identify distinct binding modes of TXNL1 that depend on the proteasome conformation and ATPase motor states. Together with biophysical and biochemical experiments, we show that the resting-state proteasome binds TXNL1 with low affinity and in variable positions on top of the Rpn11 deubiquitinase. In contrast, in the actively degrading proteasome, TXNL1 uses additional interactions for high-affinity binding, whereby its C-terminal tail covers the catalytic groove of Rpn11 and coordinates the active-site Zn^2+^. Furthermore, these cryo-EM structures of the degrading proteasome capture the ATPase hexamer in several spiral-staircase arrangements that indicate temporally asymmetric hydrolysis and conformational changes in bursts during mechanical substrate unfolding and translocation. Remarkably, we catch the proteasome in the act of unfolding the β-barrel mEos3.2 substrate while the ATPase hexamer is in a particular staircase register. Our findings advance current models for protein translocation through hexameric AAA+ motors and reveal how the proteasome uses its distinct conformational states to coordinate cofactor binding and substrate processing.

## Abstract (web summary)

The authors use time-resolved cryo-electron microscopy to reveal the interactions of the redox-active cofactor TXNL1 with the human 26S proteasome and detect ATPase motor states that indicate burst-like mechanisms for hand-over-hand substrate translocation.

## Main

The 26S proteasome unfolds and degrades hundreds of proteins to maintain proteostasis, regulate cellular processes and generate peptides with second messenger functions^1,2^. It consists of a 20S core particle (CP) capped by one or two 19S regulatory particles (RPs) that gate access to the CP proteolytic chamber^3^. The RP includes the lid and base subcomplexes. The lid comprises the noncatalytic subunits Rpn3, Rpn5–Rpn9, Rpn12 and Sem1 (human PSMD3, PSMD12, PSMD11, PSMD6, PSMD7, PSMD13, PSMD8 and SEM1, respectively), as well as the Zn^2+^-dependent deubiquitinase (DUB) Rpn11 (human PSMD14). The base subcomplex contains the heterohexameric AAA+ (ATPase associated with various cellular activities) motor with six distinct ATPase subunits in the order Rpt1, Rpt2, Rpt6, Rpt3, Rpt4 and Rpt5 (human PSMC2, PSMC1, PSMC5, PSMC4, PSMC6 and PSMC3)^4^, the large scaffolding subunit Rpn2 (human PSMD1) and the three intrinsic ubiquitin receptors, Rpn1 (human PSMD2), Rpn10 (human PSMD4) and Rpn13 (hRpn13)^5–8^. For simplicity, we will use the yeast nomenclature of proteasomal subunits that is well established for structure–function studies in the field.

Each Rpt subunit includes a N-terminal helix, which, in the assembled hexamer, forms a coiled coil with one of the neighboring Rpts, a small domain with an oligonucleotide-binding and oligosaccharide-binding fold that forms an N-terminal domain ring (N-ring), and a C-terminal ATPase domain with large and small AAA+ subdomains that constitute the ATPase motor ring^9^. The majority of substrates are targeted to the proteasome by polyubiquitin chains, which bind to a ubiquitin receptor and allow an unstructured initiation region of the substrate to enter the central channel of the ATPase motor^10–13^. A conserved pore 1 loop protrudes from each Rpt subunit into the central channel to sterically engage the substrate polypeptide^14,15^ and ATP-hydrolysis-driven conformational changes of Rpt subunits apply mechanical force for substrate unfolding and translocation into the 20S CP. Rpn11 sits above the central pore of the ATPase motor and cleaves ubiquitin chains en bloc from translocating substrates^16^.

Before substrate engagement, the 26S proteasome predominantly resides in an engagement-competent resting state (RS; also called the s1 state for the yeast proteasome), with an accessible entrance to the central channel but a restricted passage through the motor and closed CP gates^3,17^. Substrate insertion and engagement by the motor induces major conformational changes to processing states (PSs; also called non-s1 states), which are characterized by a rotated lid subcomplex and a wider channel with a coaxially aligned N-ring, ATPase ring and an open-gated 20S CP^17^. Rpn11 shifts to a central position above the Rpt hexamer that partially obstructs the channel entrance and facilitates substrate deubiquitination in a cotranslational manner^11,16,17^. Cryo-electron microscopy (cryo-EM) studies of 26S proteasomes with substrates stalled through Rpn11 inhibition or the addition of ATPγS revealed right-handed spiral-staircase arrangements of the Rpt hexamer, with 3–5 ATP-bound and 1–3 ADP-bound or nucleotide-free subunits^18–20^. In total, 4–5 Rpt subunits were engaged with the substrate polypeptide through their pore 1 loop, while 1–2 subunits were in an off position, with usually one ‘seam’ subunit located between the bottom and top subunits of the staircase. These observations led to a ‘hand-over-hand’ translocation model, in which the second-to-last bottom subunit in the staircase hydrolyzes ATP and releases the phosphate, causing the neighboring ADP-bound bottom subunit to disengage from the substrate and move as the ‘seam’ subunit to the top of the staircase. Subsequently this seam subunit exchanges ADP for ATP and reengages the substrate, which pushes the other, already substrate-engaged subunits downward in the staircase by ~ 5 Å and leads to a corresponding substrate translocation step of ~2 aa^18^. Hence, hydrolysis events appear to progress counterclockwise in the ATPase ring and phosphate release may drive the conformational changes that propel the substrate through the central channel.

Alternatively to ubiquitin modifications, substrates can be targeted to the proteasome through ubiquitin-like modifiers such as FAT10 (ubiquitin D^21^) and through interactions with proteasome-binding cofactors^22–25^. The cofactor NUB1 binds FAT10 in a partially unfolded state and delivers it together with any attached substrate moiety to the proteasome for degradation^25^. In addition to cofactors with substrate delivery functions, there are proteasome-binding cofactors with enzymatic activities, such as USP14/Ubp6, UCH37, UBE3C/HUL5, UBLCP1 and TXNL1 (refs. ^26–30^). The redox-active thioredoxin (TRX)-like protein TXNL1 is expressed in many cell types and was proposed as a nearly stoichiometric component of the human 26S proteasome. It uses its C-terminal PITH (proteasome-interacting TRX) domain for binding, while potentially reducing disulfide bonds with its N-terminal TRX domain^29,31^ but how it binds and is regulated remained unclear.

Here, we used cryo-EM and in vitro biochemical studies on actively degrading human 26S proteasomes to characterize the interaction with TXNL1. We found that TXNL1’s PITH domain binds on top of Rpn11, contacting Rpn10 and Rpn2, such that its TRX domain is positioned near the substrate entrance to the ATPase motor. Binding is low affinity and conformationally heterogeneous in RS proteasomes but becomes high affinity in substrate-engaged PSs through additional interactions of TXNL1’s C-terminal tail with Rpn11’s active-site Zn^2+^ and catalytic groove in a potentially deubiquitination-incompetent geometry. Extensive three-dimensional (3D) classification of the substrate-degrading proteasome revealed at least six distinct states of the ATPase motor with previously unknown conformations and nucleotide occupancies. These states suggest burst-phase-like ATP hydrolysis that may aid in substrate unfolding and better explain existing discrepancies between single-molecule experiments and structure-based models for hand-over-hand substrate translocation by AAA+ motors. In addition, differential interactions of proteasomal motor states with TXNL1 indicate a sophisticated coordination of degradation steps, whereby TXNL1 does not interfere with initial or cotranslocational substrate deubiquitination.

## Results

### High-resolution structures of TXNL1 bound to the human 26S proteasome

**Extended Data Fig. 1:**
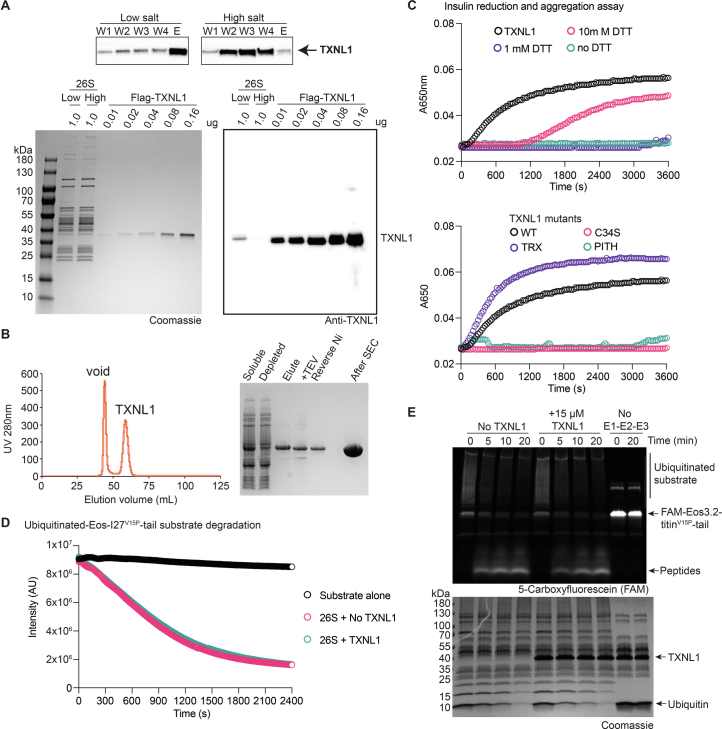
TXNL1 is redox active and co-purifies with human 26S proteasomes. **a**) Purification of TXNL1-bound and TXNL1-free human 26S proteasomes from HEK293 cells. Top: Western blots showing aliquots from the washing (W1-W4) and elution (E) steps in low-salt or high-salt buffer for HTBH-tagged proteasomes that were immobilized on streptavidin agarose. Bottom left: Coomassie-stained SDS-PAGE gel showing the separation of 1 μg human 26S proteasomes purified by size-exclusion chromatography after previous low salt or high salt washes and compared to specific concentrations of recombinant FLAG-tagged TXNL1 purified from *E. coli*. Bottom right: Western blot of the SDS-PAGE samples on the left, showing TXNL1 levels that co-purified with low-salt or high-salt washed proteasomes in comparison to recombinant His-FLAG-tagged TXNL1. Low-salt washed proteasomes contain sub-stoichiometric amounts of TXNL1, whereas TXNL1 levels for high-salt washed proteasomes are almost undetectable. **b**) Left: Elution profile for the size-exclusion chromatography (SD75 16/600) of recombinant human TXNL1 that was expressed in *E. coli* and Ni-NTA affinity purified using its His-(TEV)-FLAG tag. Right: Coomassie-stained SDS-PAGE gel with aliquots from individual stages of recombinant TXNL1 purification. **c**) Redox activity of recombinant TXNL1 (15 μM) measured by the increase in turbidity (absorbance at 600 nm) that results from the reduction and consequent aggregation of insulin (30 μM). Activities are compared to different concentrations of DTT (top) and TXNL1 mutants that contained only the N-terminal catalytic TRX domain, the C-terminal PITH domain, or the C34S mutation in the catalytic CXXC motif (bottom). **d**) Degradation of Eos-titin^V15P^-tail (5 μM) substrate by human 26S proteasome (200 nM) in the absence or presence of excess TXNL1 (15 μM), monitored by the loss of Eos fluorescence. **e**) FAM fluorescence detection (top) and Coomassie staining (bottom) of the SDS-PAGE gel with samples from the degradation of N-terminally FAM-labeled and ubiquitinated ^FAM^Eos-titin^V15P^-tail substrate (2.5 μM) by human 26S proteasomes (200 nM) in the absence and presence of excess TXNL1 (15 μM). The right 2 lanes show a negative control with non-ubiquitinated substrate (no E1, E2, E3 enzymes). Source data

We identified TXNL1 bound to endogenous proteasomes when we reprocessed a cryo-EM dataset of the human 26S proteasome during NUB1-mediated degradation of a FAT10–Eos3.2 model substrate^25^. These affinity-purified proteasomes showed substoichiometric amounts of TXNL1, whose binding was salt sensitive, such that TXNL1-free proteasomes could be prepared by high-salt washes (Extended Data Fig. 1a). To increase TXNL1 occupancy, we saturated the human proteasomes with recombinant TXNL1 that was purified from *Escherichia* *coli* (Extended Data Fig. 1b) and confirmed to be redox competent in an insulin turbidity assay (Extended Data Fig. 1c).

**Fig. 1:**
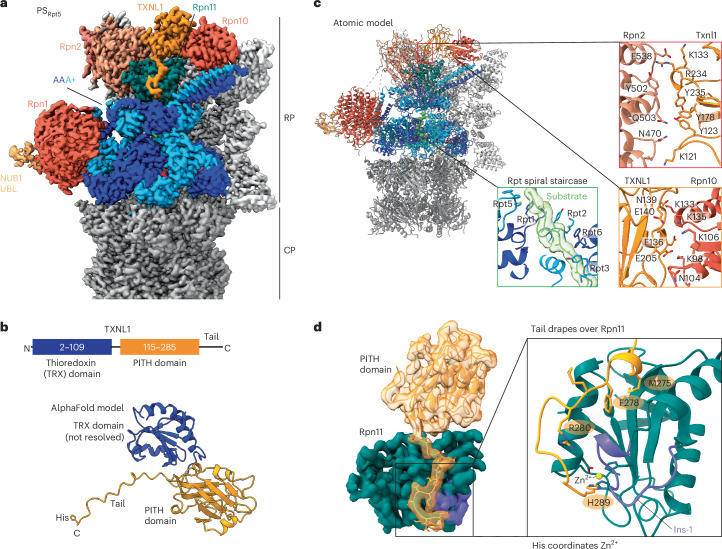
TXNL1 interacts with the substrate-degrading human 26S proteasomes and coordinates the active-site zinc of Rpn11. **a**, Density of TXNL1’s PITH domain bound to the human 26S proteasome in PS_Rpt5_ with Rpt5 at the top of the AAA+ ATPase spiral staircase and engaged with a translocating peptide during degradation of FAT10–Eos substrate delivered by NUB1. TXNL1’s PITH domain and C-terminal tail are shown in orange, the DUB Rpn11 is shown in petrol, Rpn2 is shown in salmon, Rpn1 and Rpn10’s VWA domains are shown in tomato, the AAA+ ATPase hexamer is shown in alternating dark blue and cyan, lid subunits (except Rpn11) are shown in light gray and the 20S CP is shown in dark gray. **b**, Top: schematic of TXNL1’s domain organization. Bottom: AlphaFold model of full-length TXNL1. The N-terminal TRX domain is flexibly attached to the C-terminal PITH domain and, therefore, not resolved in our EM maps. **c**, Atomic model of PS_Rpt5_ with bound TXNL1. Right: zoomed-in views depicting the interfaces of TXNL1 with Rpn2 and Rpn10, as well as the Rpt pore 1 loops forming a spiral staircase around the translocating substrate polypeptide. **d**, Left: density for the PITH domain and its C-terminal tail bound to Rpn11, with Rpn11’s Ins-1 region shown in purple. Right: zoomed-in view of TXNL1’s C-terminal tail, with hydrophobic residues (M275 and F278) pointing toward Rpn11’s catalytic groove and the C-terminal H289 coordinating the active-site Zn^2+^.

**Extended Data Fig. 2:**
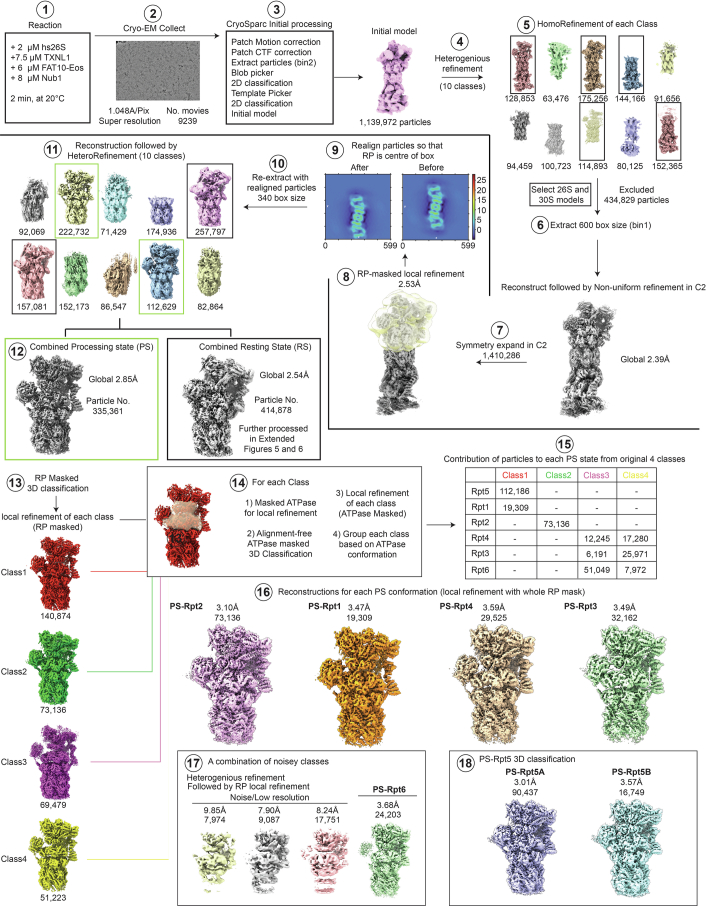
Workflow for the cryoEM structure determination of human 26S proteasomes. Shown are the sequential steps taken to process cryo-EM data from videos to final maps for the human 26S proteasome at ~ 2 min after incubation with excess Txnl1, FAT10-Eos substrate, and the NUB1 cofactor. From step 13 the workflow focuses on proteasomes in the processing states (PS), while the subsequent workflow for proteasomes in the resting state (RS) is shown in Extended Data Figs. 5 and 6.

**Extended Data Fig. 3:**
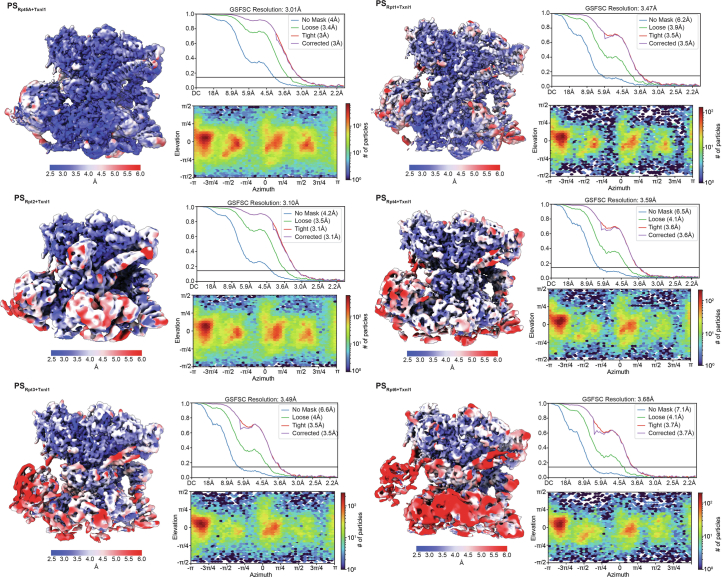
Local resolution maps for the 6 substrate-processing (PS) conformations of the human 26S proteasome in the presence of excess TXNL1. These maps resulted from the workflow shown in Extended Data Fig. 2. PS states are named based on the Rpt subunit that resides at the top of the substrate-engaged spiral staircase. Local resolutions were calculated with CryoSparc using a FSC cutoff of 0.143.

Using time-resolved cryo-EM, we investigated the conformational landscape of the substrate-degrading proteasome 2 min after mixing it with FAT10–Eos3.2, NUB1 and TXNL1, which yielded ~414,000 particles in the RS and ~335,000 particles in PSs (Extended Data Fig. 2). Deep 3D classification of the PS proteasomes resulted in at least six distinct conformations with global resolutions of 3.0–3.5 Å and different staircase registers of the Rpt hexamer (Extended Data Figs. 2 and 3 and Table 1). We refer to these PSs based on the highest substrate-engaged Rpt subunit in the staircase as PS_Rpt1_, PS_Rpt5_, PS_Rpt4_, PS_Rpt3_, PS_Rpt6_ and PS_Rpt2_, represented by ~19,300, ~112,200, ~29,500, ~32,200, ~24,200 and ~73,100 particles, respectively (Extended Data Figs. 2 and 3). They all show typical characteristics of the substrate-engaged proteasome, with a shifted and rotated lid subcomplex, the Rpt4/Rpt5 coiled coil positioned near Rpn10, a coaxial alignment of the ATPase ring with the open-gated 20S CP and the substrate polypeptide threaded through the central channel. We initially focus on the most abundant and highest-resolution (~3 Å) state, PS_Rpt5_ (Fig. 1a and Extended Data Fig. 3). In PS_Rpt5_, all five subunits from Rtp5 at the top to Rpt3 at the bottom of the staircase are substrate engaged through their pore 1 loop, while Rpt4 is the nonengaged seam subunit and at low resolution, likely because of continuous motion from the bottom to the top position. Through 3D classification focused on the ATPase hexamer, we could resolve substates A and B that differ in the position of Rpt4 (Extended Data Fig. 2) and we concentrate our discussion of structural features on state A, PS_Rpt5A_.

**Table 1:** Cryo-EM data collection, refinement and validation statistics and atomic models for the 26S proteasome with excess TXNL1 at 2 min after adding FAT10–Eos substrate and NUB1 cofactor

	PS_Rpt2_ + TXNL1 bound to partially folded Eos (RP refined)	PS_Rpt5_ + TXNL1	PS_Rpt1_ + TXNL1	PS_Rpt4_ + TXNL1	RS + TXNL1 Forward	RS + TXNL1 Backward	PS_Rpt2_ + TXNL1 (Eos resolved, AAA+ local)
EM Data Bank	EMD-47726	EMD-47719	EMD-47722	EMD-47724	EMD-47721	EMD-47720	EMD-71534
PDB	9E8O	9E8G	9E8J	9E8L	9E8I	9E8H	9PDI
**Dataset**	26S proteasome with excess TXNL1, FAT10–Eos and NUB1 incubated for 120 s						
Magnification	×81,000	×81,000	×81,000	×81,000	×81,000	×81,000	×81,000
Voltage (kV)	300	300	300	300	300	300	300
Electron exposure (e^−^ per Å^2^)	50	50	50	50	50	50	50
Defocus range (μm)	−0.7 to −2.0	−0.7 to −2.0	−0.7 to −2.0	−0.7 to −2.0	−0.7 to −2.0	−0.7 to −2.0	−0.7 to −2.0
Pixel size (Å)	1.048	1.048	1.048	1.048	1.048	1.048	1.048
Symmetry imposed	*C*_1_	*C*_1_	*C*_1_	*C*_1_	*C*_1_	*C*_1_	*C*_1_
Initial particles (*n*)	1,410,257	1,410,257	1,410,257	1,410,257	1,410,257	1,410,257	1,410,257
Final particles	73,136	95,431	19,309	29,525	62,431	53,010	73,136
Map resolution (Å) FSC threshold	3.1 0.143	3.01 0.143	3.47 0.143	3.59 0.143	2.87 0.143	2.9 0.143	2.98 0.143
**Refinement**							
Model resolution (Å) FSC threshold	3.1 0.5	2.9 0.5	3.4 0.5	3.5 0.5	2.8 0.5	2.8 0.5	2.9 0.5
Map sharpening *B* factor (Å^2^) (determined)	−50.5	−50.4	−27	−29.9	−47.2	−45.3	−49.6
Initial model used (PDB)	8USC	8USC	8USC	8USC	8USC	8USC	8USC
Nonhydrogen atoms	83,181	82,194	70,858	70,522	71,304	71,304	33,552
Residues	10,532	10,415	8,967	8,939	8,986	8,986	4,241
Ligands	Zn^2+^: 1 Mg^2+^: 3 ATP: 2 ADP: 4 CR8: 1	Zn^2+^: 1 Mg^2+^: 5 ATP: 3 ADP: 3 UNK: 12	Zn^2+^: 1 Mg^2+^: 5 ATP: 4 ADP: 2 UNK: 10	Zn^2+^: 1 Mg^2+^: 3 ATP: 2 ADP: 4 UNK: 13	Zn^2+^:1 Mg^2+^:6 ATP:5 ADP:1	Zn^2+^: 1 Mg^2+^: 6 ATP: 5 ADP: 1	Zn^2+^: 1 Mg^2+^: 3 ATP: 2 ADP: 4 UNK: 22
*B* factors (Å^2^) (min/max/mean)							
Protein	15.62/408.71/134.25	28.19/207.68/92.08	30.99/177.39/71.82	84.71/567.71/186.99	9.64/428.56/66.08	15.22/376.19/65.50	57.32/299.16/131.17
Ligand	80.74/181.62/114.17	43.13/135.11/79.05	36.17/70.91/49.10	124.90/183.00/155.97	26.88/81.49/40.45	28.47/84.97/39.91	62.24/188.08/121.97
Root-mean-square deviation							
Bond lengths (Å)	0.006	0.005	0.006	0.005	0.004	0.004	0.005
Bond angles (°)	1.122	0.97	1.028	1.116	0.95	0.945	1.075
MolProbity	2.83	1.91	3.41	2.55	1.97	1.98	2.56
Clashscore	23.77	61.18	69.78	19.63	11.89	11.58	17.98
Poor rotamers (%)	4.53	5.17	5.61	2.69	2.36	2.45	3.09
Ramachandran plot							
Favored (%)	92.36	94.09	90.72	93.24	97.53	97.50	93.23
Allowed (%)	7.41	5.49	8.80	6.33	2.46	2.47	6.55
Disallowed (%)	0.23	0.42	0.47	0.43	0.01	0.03	0.21

**Extended Data Fig. 4:**
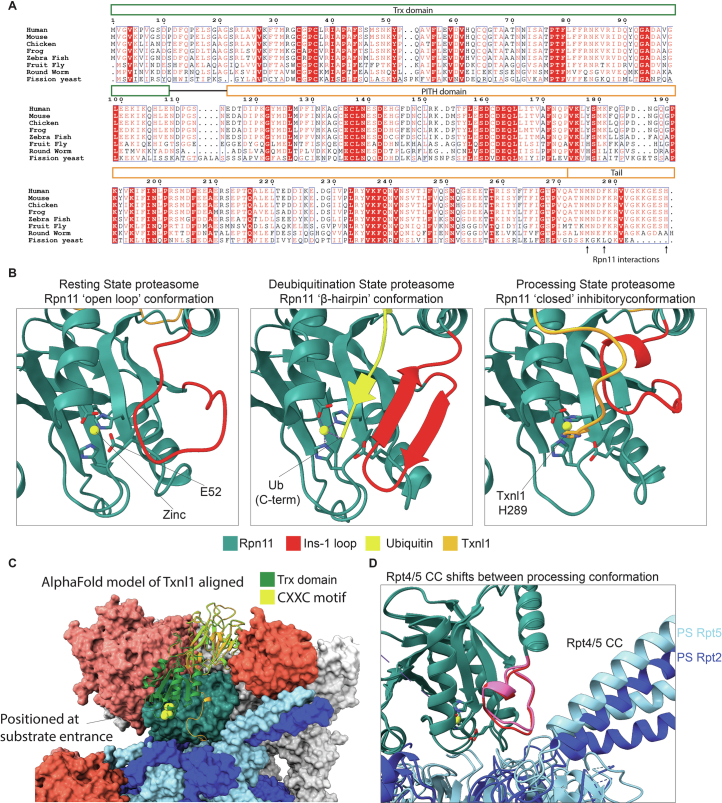
TXNL1 interacts with Rpn11 in a conformation-specific manner using its conserved C-terminal tail. **a**) Sequence alignment of TXNL1 from uniport sequences using ENDscript at default settings: *Homo sapiens* (Q43396), *Mus musculus* (Q8CDN6), *Gallus gallus* (A0A1D5NUH1), *Xenopus tropicalis* (F6RTD9), *Danio rerio* (F6NTA0), *Drosophila melanogaster* (Q9VRP3), *Caenorhabditis elegans* (G5EES9) and *Schizosaccharomyces pombe* (Q9USR1). Red underlay shows conserved residues and blue boxes indicate similarity in amino acid identity. Residues in the C-terminal tail that mediate the interaction with Rpn11, including the terminal His, are highly conserved, except for fission yeast. **b**) Atomic models of Rpn11 with the Insert-1 (Ins-1) region in three conformations. The open loop conformation is found in resting-state proteasomes, the deubiquitination conformation with a β-hairpin is adopted upon ubiquitin binding to Rpn11, and the inhibitory, closed conformation is observed substrate-processing proteasomes. The three models are derived from our structure for the resting state RS.1 with TXNL1 in the forward orientation, the crystal structure of ubiquitin-bound Rpn11-Rpn8 (PDB ID: 5U4P), and our structure of the substrate-processing state PS_Rpt5_, respectively. **c**) Space filling atomic model of PS_Rpt5_ with the ribbon-represented PITH domain of TXNL1 in orange. The AlphaFold model for full-length TXNL1 is aligned by its PITH domain (light green) with the PITH domain in our structure and shows the TRX domain (dark green) with its catalytic CXXC motif (yellow spheres) close to the entrance of the ATPase motor. **d**) Overlay of the atomic models for PS_Rpt5_ (Rpts in cyan) and PS_Rpt2_ (Rpts in dark blue), aligned by Rpn11 (petrol), shows a movement of the Rpt4/Rpt5 coiled coil that significantly increases the gap to the Ins-1 loop (pink for PS_Rpt5_, red for PS_Rpt2_).

Extra density above Rpn11 could be unambiguously assigned to the PITH domain of TXNL1 (Fig. 1a,b), which forms predominantly ionic contacts with Rpn2 and Rpn10’s VWA (von Willebrand factor type A) domain (Fig. 1c), explaining its salt sensitivity. The N-terminal catalytic TRX domain could not be resolved (Fig. 1a), likely because of its flexible linkage to the PITH domain; however, aligning AlphaFold models for full-length TXNL1 with our structures places the TRX domain near the substrate entrance (Extended Data Fig. 4c). The C-terminal tail of TXNL1 draped over Rpn11’s hydrophobic catalytic groove and the insert-1 (Ins-1) loop, which has important roles in binding the C terminus of ubiquitin and regulating access to the deubiquitination active site (Fig. 1d).

### TXNL1 coordinates the active-site zinc of Rpn11 but does not inhibit deubiquitination

TXNL1 interacts with the catalytic groove of Rpn11 through its hydrophobic tail residues F278 and M275 (Fig. 1d). Interestingly, the C terminus of this tail reaches into Rpn11’s active site and uses a conserved terminal histidine to coordinate the catalytic Zn^2+^ (Fig. 1d and Extended Data Fig. 4a).

The Ins-1 region of Rpn11 is a critical regulatory segment with at least three distinct states described so far: an ‘open’ loop conformation in the RS proteasome, an active β-hairpin conformation where Ins-1 forms a small β-sheet with the C terminus of ubiquitin to position it for cleavage and a ‘closed’ or inhibitory conformation typically found for ubiquitin-free Rpn11 in PS proteasomes (Extended Data Fig. 4b)^16,18,19,32^. The specific conformation and interactions of TXNL1’s C-terminal tail are compatible only with the closed state of Ins-1, whereas the ubiquitin-bound state would lead to steric clashes and the open state would be unable to form the necessary contacts. TXNL1’s tail may, thus, bind the catalytic groove of Rpn11 only on substrate-processing proteasomes when no deubiquitination is occurring.

**Extended Data Fig. 5:**
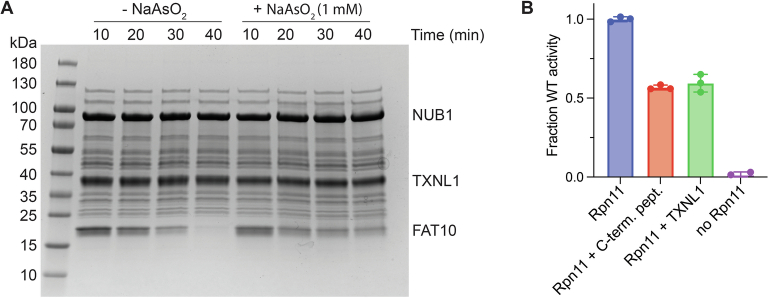
TXNL1 interacts with the catalytic groove of Rpn11, but does not get degraded by the 26S proteasome in isolation, even in the presence of arsenite. **a**) Arsenite treatment perturbs FAT10 degradation, but does not induce TXNL1 degradation *in vitro*. Coomassie-stained SDS-PAGE gel showing time points for the degradation of FAT10/NUB1 (5 μM) by the 26S proteasome (100 nM) in the presence of TXNL1 (5 μM) and the absence or presence of arsenite (1 mM). **b**) The ubiquitin-cleavage activity of the isolated Rpn11/Rpn8 heterodimer is inhibited by TXNL1 or a peptide TNMNDFKRVVGKKGESH representing the last 17 residues of TXNL1’s C-terminus. Shown are the relative activities in ubiquitin-Lys-TAMRA cleavage by Rpn11/Rpn8 in the presence of 633 μM TXNL1 or C-terminal peptide compared to Rpn11/Rpn8 alone (shown are the mean +/− SD for n = 3 technical replicates). Source data

Previous studies suggest that polyubiquitinated substrates use a distal ubiquitin in the chain to interact with a proteasomal receptor; before the most proximal ubiquitin binds to Rpn11, the substrate’s flexible initiation region inserts into the central channel and engagement by the ATPase motor triggers the conformational switch from resting to PSs that allow subsequent cotranslational deubiquitination^11,19,33,34^. To test whether TXNL1 inhibits deubiquitination, we prepared a model substrate (Eos–I27^V15P^-tail) in which Eos3.2 was fused to the titin I27 domain with a destabilizing V15P substitution and a long flexible C-terminal tail containing a single lysine residue for Rsp5-mediated polyubiquitination. Multiple-turnover degradation monitored by the loss in Eos fluorescence showed identical rates with and without excess TXNL1 (0.54 ± 0.06 min^−1^; Extended Data Fig. 1d). Similarly, monitoring substrate deubiquitination and peptide product formation by SDS–PAGE with an N-terminally fluorescein-labeled Eos–I27^V15P^-tail substrate revealed no obvious effect of TXNL1 on ubiquitin-dependent degradation (Extended Data Fig. 1e), suggesting that TXNL1’s interaction with Rpn11 does not interfere with the removal of the affinity-conferring ubiquitin chain from a substrate upon motor engagement. All proteasomes were pretreated with ubiquitin-propargylamine (Ub-PRG) to inhibit cysteine-dependent DUBs and rule out their potential compensation for Rpn11 activity. In contrast to the substrate-degrading proteasome, isolated Rpn11 was inhibited by high concentrations of TXNL1 or a TXNL1 C-terminal peptide (Extended Data Fig. 5a), consistent with a context-dependent, conformationally selective binding of TXNL1.

Despite in vivo data indicating proteasome-dependent TXNL1 degradation upon arsenite treatment of mammalian cells^31^, TXNL1 was not degraded in vitro (Extended Data Fig. 1e), even in the presence of arsenite (Extended Data Fig. 5b). This rules out simple mechanisms by which arsenite induces TXNL1 unfolding to an engageable state or the disulfide crosslinking to a cysteine-containing substrate for codegradation.

### TXNL1 forms low-affinity interactions with RS proteasomes

**Fig. 2:**
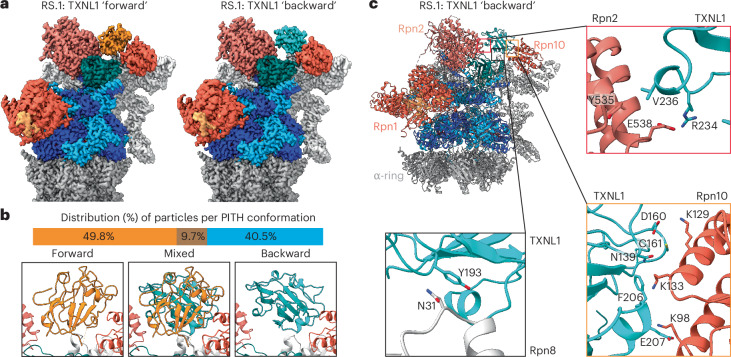
TXNL1 binds the RS proteasome in multiple conformations through contacts with Rpn2 and Rpn10’s VWA domain. **a**, Densities of the RS.1 26S proteasome bound to TXNL1’s PITH domain in the forward (left; PITH shown in orange) and backward (right; PITH shown in cyan) conformations. The forward position is identical to the location of the PITH domain on PS proteasomes, whereas the backward conformation is rotated by ~90°. RS.1 refers to a particular class of RS proteasomes that differ from RS.2 through a slight shift in the RP (Extended Data Fig. 7). **b**, Particle distribution of RS proteasomes with TXNL1 bound in the forward, backward or mixed conformations. Mixed conformations represent intermediate states because of PITH domain motions or particles with ambiguous probabilities of belonging to either conformation. **c**, Top left: atomic model of the RS.1 proteasome with the PITH domain (cyan) in the backward conformation. Right and bottom: zoomed-in views of specific interactions between the PITH domain and Rpn2, Rpn10’s VWA domain and Rpn8. The contacts with Rpn2 and Rpn10 strongly rely on ion pairs, whereas a polar–*π* interaction is at the center of the interface with Rpn8.

**Extended Data Fig. 6:**
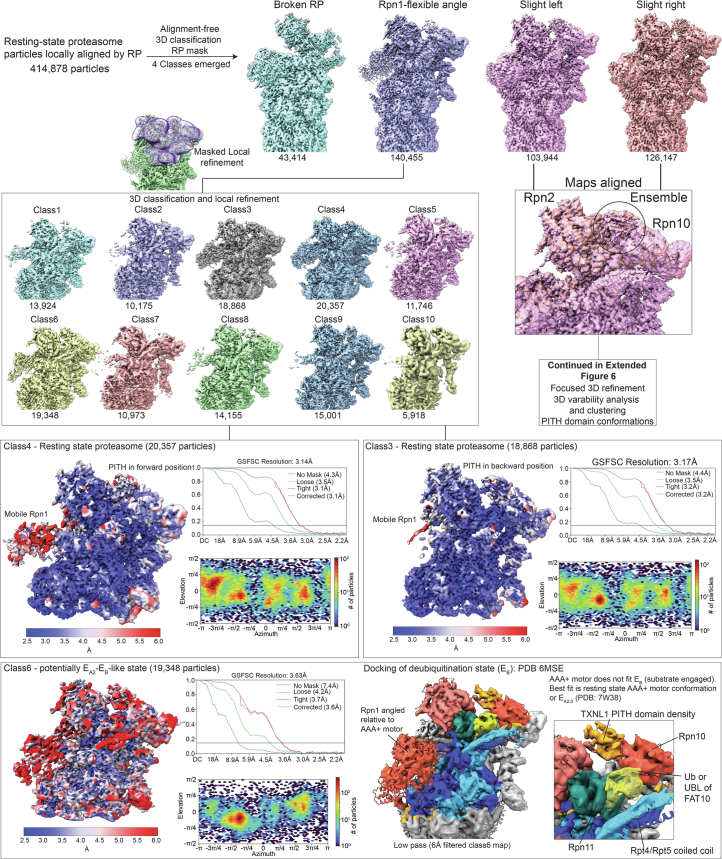
Further cryo-EM data processing of resting-state (RS) proteasomes in continuation from Extended Data Fig. 2. Classification leads to the emergence of 4 classes. Two of these classes, called ‘Slight left’ and ‘Slight right’ based on the position of the regulatory particle, show an ensemble of PITH domain densities and were further processed as described in Extended Data Fig. 7. Further classification of class 2 with variability in the Rpn1 subunit revealed 10 additional classes at moderate resolution. Three of them, class 4, class 3, and class 6 are shown in more detail below. Class 4 has TXNL1’s PITH domain in the forward conformation, whereas class 3 shows the PITH domain oriented backwards. Class 6 represents a unique class with extra density on Rpn11 that may originate from ubiquitin or a ubiquitin-like domain, for instance of FAT10. A human proteasome structure (PDB: 6MSE) with ubiquitin-bound Rpn11 was used for comparison, to color the class 6 map, and show similarities.

**Extended Data Fig. 7:**
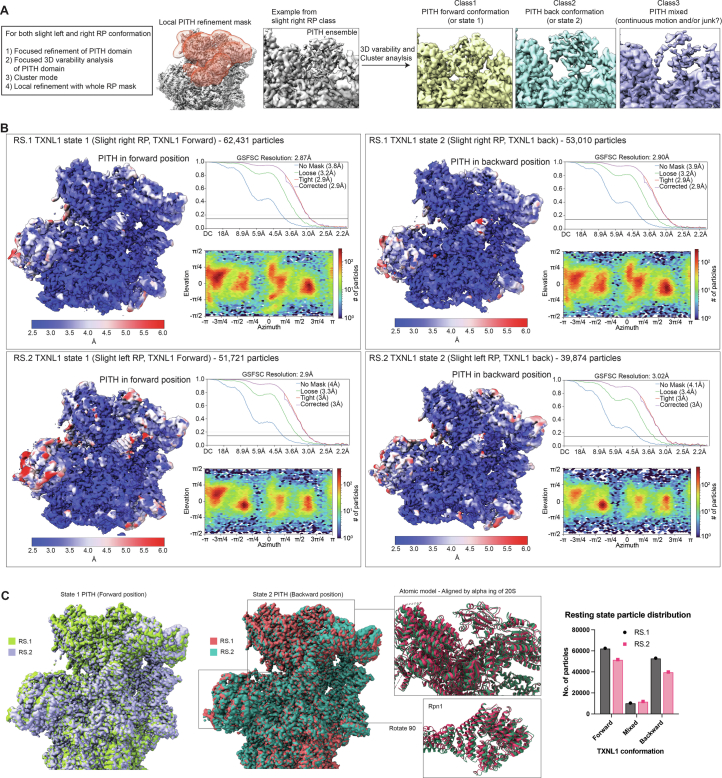
Further cryo-EM data processing of resting-state (RS) proteasomes with bound TXNL1 in continuation from Extended Data Fig. 6. **a**) ‘Slight left’ and ‘Slight right’ particles were processed separately. Particle stacks were aligned (local refinement in CryoSparc) with a local mask surrounding the PITH domain, Rpn2, Rpn10, Rpn11, and the N-ring of the Rpt hexamer. CryoSparc 3D variability analysis of aligned particles with the local mask, followed by 3D cluster analysis, allowed grouping of particles into two distinct conformations, forward and backward with respect to the PITH domain position. A third group of particles showed the PITH domain in variable positions and could not be assigned to a particular state, suggesting that the PITH domain has continuous motion between forward and backward orientations. **b**) Local refinement with a mask surrounding the whole RP resulted in 4 distinct conformations defined by a slight shift in the RP and the two orientations of the PITH domain: RS.1 TXNL1 Forward (RS.1 state 1), RS.1 TXNL1 Backward (RS.1 state 2), RS.2 TXNL1 Forward (RS.2 state 1), and RS.2 TXNL1 Backward (RS.2 state 2). Each structure was resolved to high resolution, as demonstrated by the maps colored according to estimations of local resolutions, calculated with CryoSparc using a 0.143 FSC cutoff. **c**) Alignments of the atomic models for RS.1 and RS.2, states 1 and 2, highlight the shift in the RP, in addition to variations in Rpn1’s position. Right: Particle numbers for each conformational state that were used to calculate the percentages of proteasomes with TXNL1 in the forward, backward, or mixed orientations shown in Fig. 2b. Source data

Interestingly, although our cryo-EM dataset showed a higher number of RS proteasomes, TXNL1’s tail was not detected interacting with Rpn11, likely because of its incompatibility with Rpn11’s Ins-1 loop in the open state (Fig. 2). Instead, the PITH domain adopted an ensemble of conformations between Rpn2 and Rpn10 (Fig. 2 and Extended Data Figs. 6, and 7), whose classification resulted in RS.1 and RS.2 states, with minor lid movement and no changes for the ATPase motor (Extended Data Fig. 7). Local refinement of the PITH domain and 3D variability analyses for both RS.1 and RS.2 allowed us to separate two major PITH domain states that we term forward (49.8 % of particles) and backward (40.5% of particles) according to the PITH domain rotation (Fig. 2a,b and Extended Data Fig. 7), along with a smaller number of particles showing the PITH domain in intermediate positions. Thus, in the absence of C-terminal tail binding to Rpn11, the PITH domain appears to form a ‘blurry’ interaction with the RS proteasome, using just a few contact points on Rpn2 and Rpn10’s VWA domain to swivel between forward and backward positions by ~91°. The forward position resembles the TXNL1 conformation we observed in the PS proteasomes, except that the C-terminal tail does not contact Rpn11 and is, therefore, not resolved (Figs. 2a and 1a). The backward conformation uses overlapping sites on Rpn2 and Rpn10 but distinct contact points on the PITH domain and again no C-terminal tail (Fig. 2c). These alternative orientations and their poorly resolved intermediate states provide an interesting example for ‘fuzzy’ multimode interactions of a cofactor with a large protein complex. The 3D classification of a separate RS, where Rpn1 is mobile and at lower resolutions, also highlighted that forward and backward conformations were populated (Extended Data Fig. 6).

The backward conformation appears compatible with deubiquitination. Indeed, for a proteasome conformation similar to the E_A2_ or E_B_ states, which were previously proposed to reflect substrate deubiquitination^19,20^, we observed TXNL1 at low resolution in the backward orientation and, in addition, detected a low-resolution density bound to Rpn11 that may represent copurified ubiquitin or a UBL domain of the FAT10 substrate (Extended Data Fig. 6). Similar to the E_B_ state, these proteasomes show Rpn1 at a kinked angle relative to the ATPase ring, yet the ATPase ring has no translocating polypeptide in the channel and is in a true RS conformation, like E_A2_. However, in contrast to the E_A2_ state that was reported to have two ADPs bound to Rpt6 and Rpt5 across from each other in the hexamer, we observed five subunits bound to ATP and only the seam subunit Rpt6 bound to ADP (Extended Data Fig. 6). The backward conformation of TXNL1 is, hence, exclusively found in RS proteasomes, whereas the forward conformation is populated in both RS and actively degrading proteasomes, with the latter showing additional interactions of TXNL1’s C-terminal tail with Rpn11’s catalytic groove and active-site Zn^2+^.

### TXNL1 binds to actively degrading proteasomes with high affinity

**Fig. 3:**
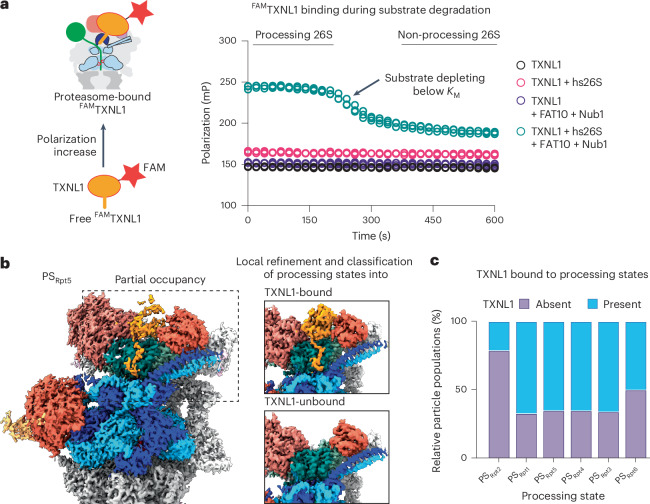
TXNL1 specifically interacts with PS 26S proteasomes. **a**, Proteasome binding and dissociation of N-terminally FAM-labeled TXNL1 (50 nM) was measured by changes in FP after incubation with human 26S proteasome (500 nM) in the absence or presence of FAT10 substrate (10 μM) and the NUB1 cofactor (12 μM). **b**, Time-resolved cryo-EM of actively degrading proteasomes with substoichiometric amounts of TXNL1 reveals TXNL1’s binding preference for PSs. Left: EM density of the proteasome in the PS_Rpt5_ conformation shows partial occupancy with TXNL1’s PITH domain (orange). Right: zoomed-in views of example density maps from particles that were sorted into TXNL1-bound (top) and TXNL1-unbound (bottom) after local 3D classification and refinement of the PITH domain density. **c**, Fractions of proteasome particles with and without bound TXNL1 as a function of the PS conformation. *K*_M_, Michealis constant. Source data

**Extended Data Fig. 8:**
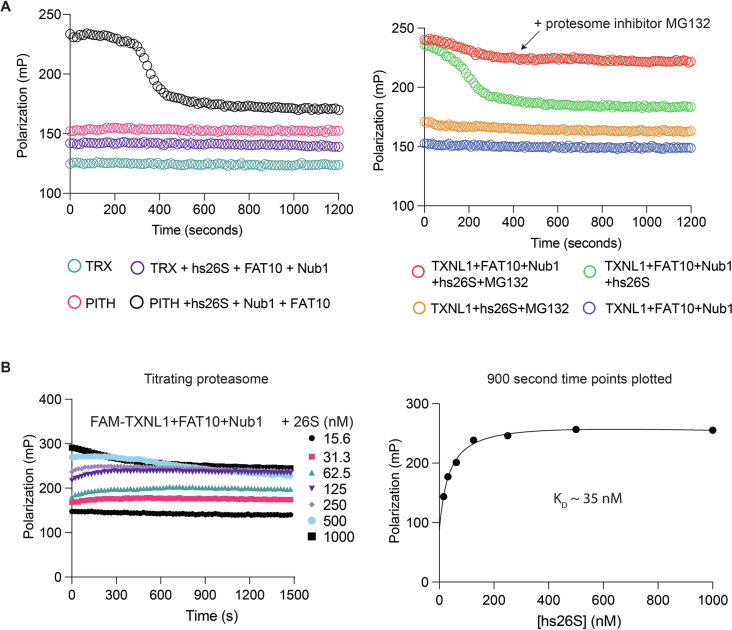
TXNL1 dissociates from proteasomes as they transition back from processing to the resting state, but remains bound when degradation is stalled. **a**) Left: TXNL1’s PITH domain is necessary and sufficient for the conformational selective binding to actively degrading 26S proteasomes. Shown are representative traces for the fluorescence polarization of FAM-labeled TRX domain (50 nM) or PITH domain (50 nM) that was incubated in isolation or with human 26S proteasome (500 nM) in the presence of 10 μM FAT10 substrate and 12 μM NUB1 cofactor. Right: Proteasomes whose proteolytic cleavage of the FAT10 substrate was inhibited by MG132 and which were therefore stalled during substrate translocation interact stably with TXNL1. Shown are the fluorescence polarization time courses for N-terminally FAM-labeled full-length ^FAM^TXNL1 (50 nM) that was incubated with FAT10 (5 μM), NUB1 (6 μM), and human 26S proteasome (500 nM) in the absence or presence of MG132 (20 μM), as indicated. **b**) Titration of proteasomes that were stalled during substrate degradation through MG132 inhibition of proteolysis reveals the TXNL1 binding affinity for processing-state proteasomes. Left: Shown are the fluorescence polarization traces for ^FAM^TXNL1 (50 nM) incubated with human 26S proteasome at indicated concentrations in the presence of FAT10 substrate (5 μM) and NUB1 cofactor (6 μM). Right: ^FAM^TXNL1 binding curve derived from the 900 s time points of the polarization traces shown on the left indicates an affinity of *K*_*D*_ ~ 35 nM. Source data

To test whether TXNL1 preferentially binds PS proteasomes, we measured fluorescence polarization (FP) of N-terminally FAM-labeled TXNL1 mixed with excess human proteasome in the absence or presence of FAT10 substrate and NUB1 cofactor. RS proteasomes in the absence of substrate produced low FP, whereas actively degrading proteasomes showed high FP that decayed as the substrate was consumed and the conformational equilibrium shifted back to the RS, indicating TXNL1 dissociation (Fig. 3a). The TRX domain deletion (PITH) showed high FP that decayed similar to full-length TXNL1, while the PITH domain deletion (TRX) had low FP, confirming that TXNL1 binding is solely dictated by the Rpn11-interacting PITH domain (Extended Data Fig. 8a). Blocking CP with an inhibitor to internally accumulate substrate and stall translocation yielded a persistently high FP signal (Extended Data Fig. 8a), indicating that TXNL1 binds tightly to proteasomes trapped in PSs. Titration of these stalled complexes revealed a dissociation constant *K*_D_ of ~35 nM (Extended Data Fig. 8b), whereas TXNL1 titrations we performed with RS proteasomes for cryo-EM structure determination let us estimate a *K*_D_ in the tens of micromolar range, at least two orders of magnitude higher than for the PSs. Hence, TXNL1 binds PS proteasomes with high affinity but reverts to low-affinity fuzzy binding and dissociates when the proteasome switches back to the resting conformation.

### Time-resolved cryo-EM reveals conformation-specific TXNL1-binding preference

**Extended Data Fig. 9:**
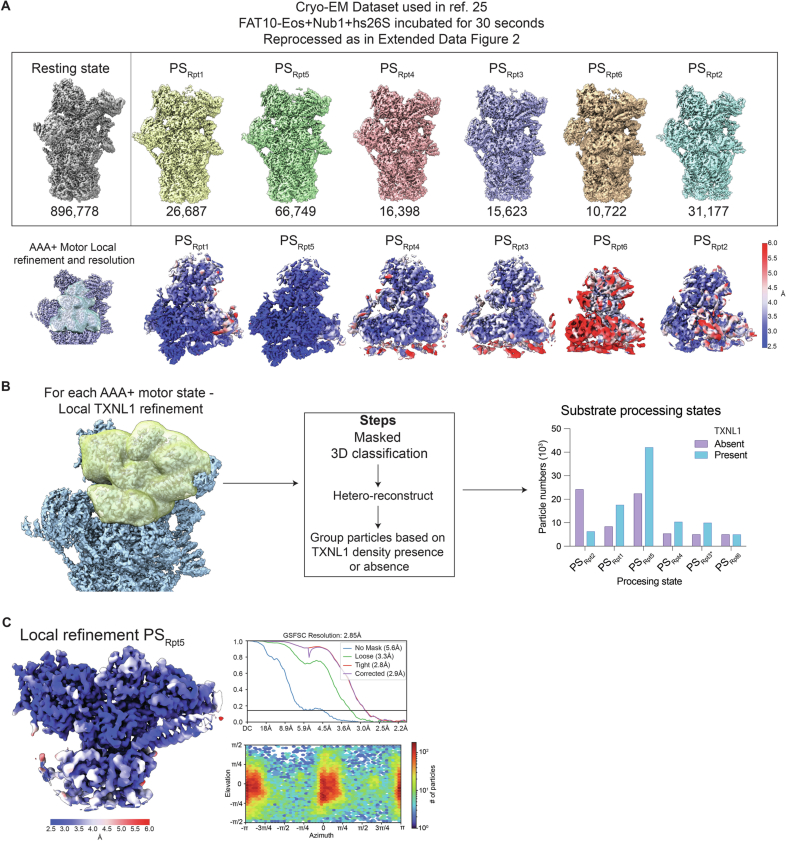
Cryo-EM data processing for the human 26S proteasomes 30 s after incubation with FAT10 substrate and NUB1 cofactor. **a**) Data were processed as shown in the workflow in Extended Data Fig. 2. Top: Shown are the unsharpened maps and particle numbers for each state. Bottom: To improve resolution of the AAA+ ATPase domains a local mask was generated for the Rpt hexamer and Rpn11 and local refinement was used. Maps show local resolutions of the Rpt subunits and Rpn11 for each processing state. **b**) Human proteasomes used in this dataset were purified from HEK293 cells under low-salt conditions and therefore contain sub-stoichiometric amounts of bound TXNL1. Extensive local 3D classification focused on TXNL1 for each processing state allowed separation of particles with and without bound TXNL1. Briefly particles were locally aligned by a mask surrounding TXNL1’s PITH domain and subjected to 3D classification with a filtered resolution of 10 Å. Each class was reconstructed and refined to group particles based on the presence or absence of PITH density, and the particles numbers for PITH-bound and -unbound proteasomes are plotted for each processing state (right). **c**) Local resolution of locally refined PS_Rpt5_ after removing unbound TXNL1 particles. Source data

When further processing a previously collected dataset for the human proteasome prepared 30 s after initiating degradation of a FAT10–mEos3.2 substrate, we observed a large number of RS particles (77.3%) without TXNL1 density, consistent with the substoichiometric amount of copurified TXNL1 and its low affinity for the RS. Substrate-degrading proteasomes were detected in the same six staircase ATPase states as for the 2 min time point (Extended Data Fig. 9a), with PS_Rpt1_, PS_Rpt5_, PS_Rpt4_, PS_Rpt3_, PS_Rpt6_ and PS_Rpt2_ represented by ~26,700, ~66,800, ~16,400, ~15,600, ~10,700 and ~31,200 particles, respectively, and refined to moderate global resolutions of 3–4.5 Å (Fig. 3b and Extended Data Fig. 9a). Local refinement of masked Rpts improved the ATPase hexamer resolution for model building and interpretation of nucleotides, as discussed below (Extended Data Fig. 9a), and focused alignments on the PITH domain allowed us to achieve high resolution (~2.8 Å for PS_Rpt5_) and the classification of PITH-bound and PITH-unbound states for each ATPase conformation (Fig. 3b,c and Extended Data Fig. 9b,c). We noted a clear bias of PS_Rpt2_ for being TXNL1-free (Fig. 3c and Extended Data Fig. 9b). Interestingly, PS_Rpt2_ exhibited a larger gap between the coiled coil of Rpt4/5 and Rpn11’s Ins-1 region (Extended Data Fig. 4d), perhaps increasing Ins-1 dynamics and destabilizing TXNL1 tail contacts, similar to the RS. It is tempting to speculate that the Ins-1 conformation in PS_Rpt2_ is less inhibitory for deubiquitination and more readily transitions to the active hairpin state that forms a β-sheet with the C terminus of ubiquitin. PS_Rpt2_ may, therefore, represent a PS that is suited for the removal of ubiquitin chains during substrate translocation or unfolding.

Overall, the structures of the degrading human 26S proteasome in the presence of substoichiometric TXNL1 further support our findings that TXNL1 forms high-affinity interactions only with proteasome PSs, except for PS_Rpt2_, in which low-affinity binding may prevent TXNL1’s interference with cotranslocational deubiquitination.

### Capturing an unfolding intermediate of the Eos substrate

**Fig. 4:**
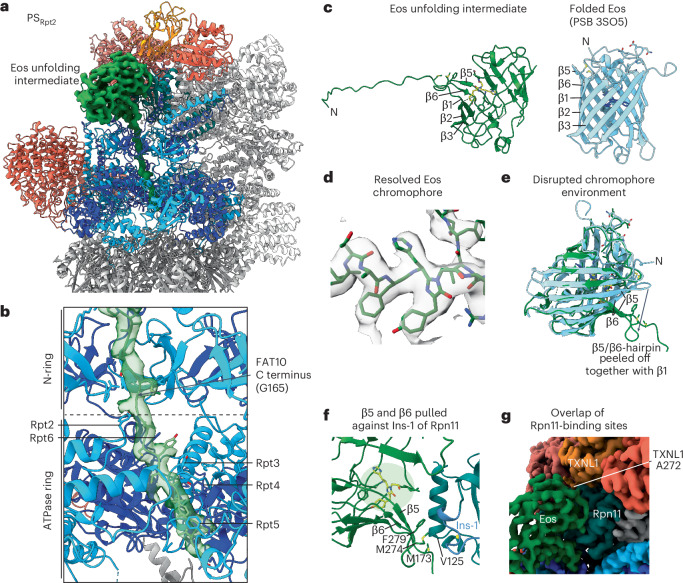
Visualizing a partially unfolded Eos intermediate during active degradation by the 26S proteasome. **a**, Atomic model of the human proteasome in the PS_Rpt2_ state during degradation of the FAT10–Eos model substrate. EM density (green) is shown for the substrate, with the partially unraveled β-barrel of Eos pulled against Rpn11 and a translocating polypeptide spanning the central channel of the ATPase motor. **b**, Focus on the Rpt hexamer with the EM density and atomic model (green) shown for the translocating substrate, which is engaged by a staircase of ATPase domains. The substrate density is at high enough resolution to identify the C-terminal portion of FAT10 inside the ATPase ring. **c**, Comparison between the partially unfolded Eos intermediate (left, green) and the crystal structure of folded Eos (right, cyan; PDB 3S05), with the chromophore shown in yellow and dark blue, respectively. The intermediate has β1 and the β2–β3 and β5–β6 hairpins partially pulled off from the β-barrel. **d**, EM density and atomic model for the chromophore in partially unfolded Eos. **e**, Overlay of the folded (cyan) and partially unfolded Eos (green) show the disruption of the chromophore environment that likely leads to a loss of fluorescence. **f**, Hydrophobic residues in β1 and the β5–β6 hairpin that normally face the Eos hydrophobic core interact with Rpn11 near the catalytic groove and the Ins-1 loop. **g**, EM density for proteasomes with bound TXNL1 and the Eos unfolding intermediate show an overlap of binding sites for Eos and TXNL1’s C-terminal tail on Rpn11.

Using the dataset for proteasomes with saturating TXNL1 at 2 min after initiating FAT10 substrate degradation, we visualized the PS_Rpt2_ conformation with an Eos unfolding intermediate above the motor entrance and braced against Rpn11 (Fig. 4a). Despite lower global resolution for Eos, we could reliably position the β-barrel. Sharpened maps, with better side-chain resolutions, allowed us to model the linker N terminus of Eos and the following 11 C-terminal residues of FAT10, as they span through the N-ring and AAA+ staircase (Fig. 4a,b). The Eos domain is partially unfolded, with its FAT10-attached β1 strand extracted from the β-barrel and the neighboring β2–β3 and β5–β6 hairpins strongly distorted (Fig. 4c). β5 and β6 apparently maintain their hydrogen bonds as a hairpin, despite being bent to parallel the extracted β1 strand (Fig. 4e,f). We were also able to model the cyclic chromophore, formed from residues H227, Y228 and G229 (Fig. 4d) but surrounding residues could not be modeled because of the strong distortion of the chromophore’s environment (Fig. 4e). It is, therefore, likely that Eos loses its fluorescence while forming this intermediate, whose subsequent further unfolding appears to be rate-limiting for FAT10–Eos degradation. Indeed, we previously found that the Eos fluorescence decayed with *τ* = 18 s during single-turnover degradation but *τ* = 50 s under multiple-turnover conditions, likely determined by unfolding of the nonfluorescent intermediated^25^. Hydrophobic residues in β1, β5 and β6 that are normally part of the hydrophobic core interact with the surface of Rpn11. In particular, M173, M274 and F279 form hydrophobic interactions with Rpn11’s Ins-1 region and V125 of helix α3 (Fig. 4f), stabilizing the intermediate in a position on Rpn11 that overlaps with the binding site for TXNL1’s C-terminal tail (Fig. 4g). Future studies will have to address whether this Rpn11 surface is generally used to pull substrates against and potentially assist in their mechanical unfolding. Although PS_Rpt2_ is not the most abundant PS (Extended Data Fig. 2), it is the only one showing an unfolding intermediate. Curiously, previous structural studies of yeast and human proteasomes with stalled substrates also captured Rpt2 at the top of the staircase^18,20^, suggesting that this motor register may have important roles during substrate unfolding or deubiquitination. Importantly, PS_Rpt2_ proteasomes without the Eos unfolding intermediate also showed a major reduction in TXNL1 binding (Fig. 3c), indicating that high-affinity TXNL1 interaction is disfavored by the PS_Rpt2_ conformation itself and not simply prevented by steric hindrance with the Eos unfolding intermediate pulled against Rpn11.

### Asymmetric ATP hydrolysis in the Rpt hexamer

**Fig. 5:**
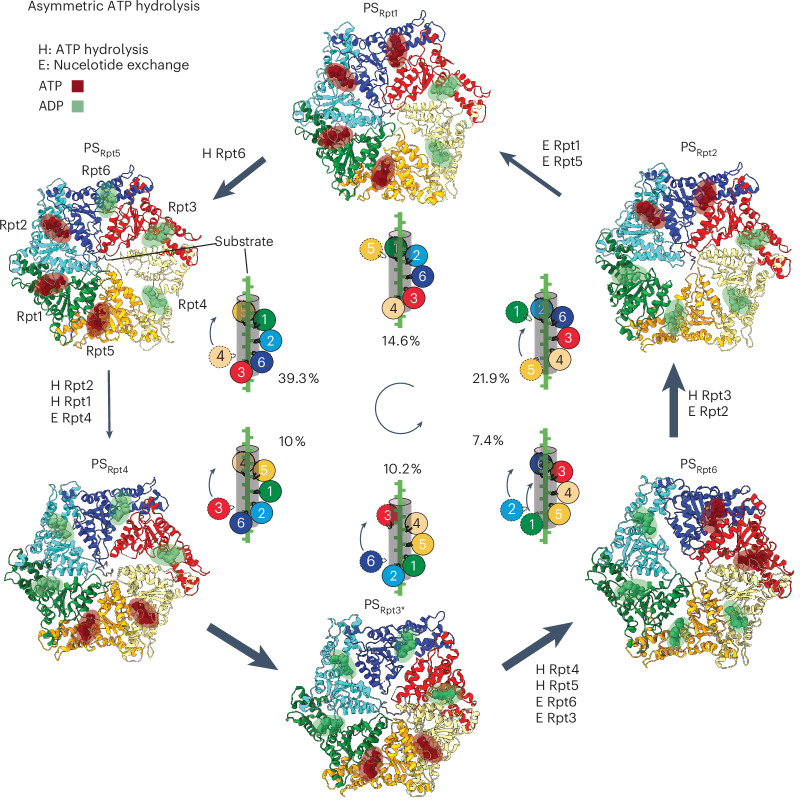
Conformational landscape of the proteasomal AAA+ motor with six distinct spiral-staircase registers during asymmetric ATP hydrolysis and active substrate processing. Atomic models show the AAA+ ATPase domains in different colors with the engaged substrate in the central channel, bound ATP depicted in red and ADP in green. States are arranged in a cycle according to the plausible order of events and transitions. The schematics depict the spiral-staircase arrangements and Rpt contacts with the substrate. Calculated cryo-EM particle distributions (as percentages) demonstrate asymmetries in the lifetime of individual PSs and the kinetics of their transitions, which is highlighted by different size arrows. For each transition the ATP hydrolysis (H) and ADP-to-ATP exchange (E) events are indicated.

**Extended Data Fig. 10:**
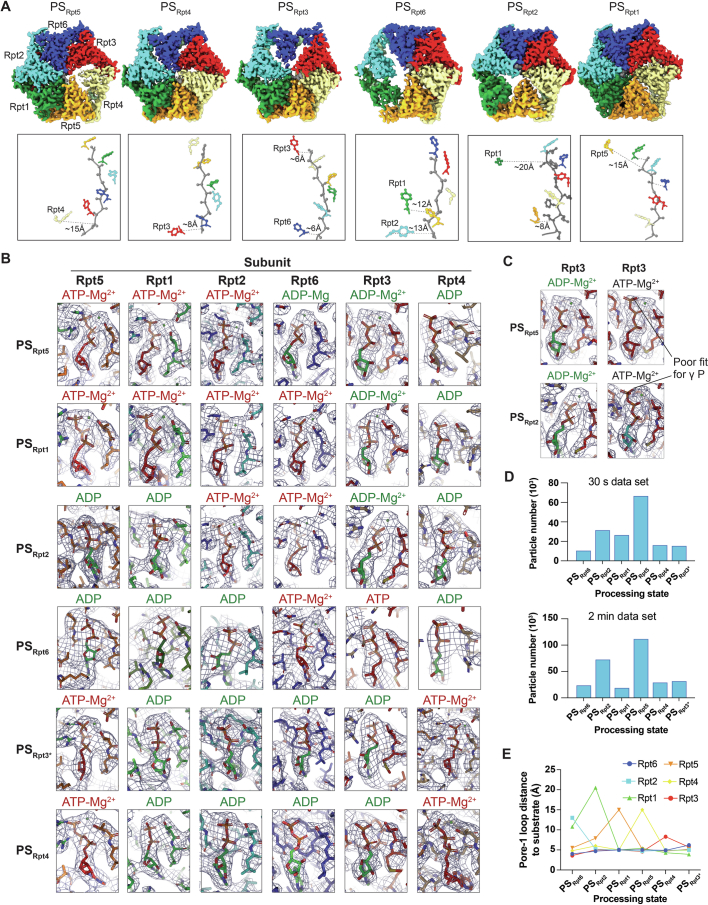
Comparisons of the six discrete substrate-processing states for the human 26S proteasome regarding spiral-staircase arrangements, pore-loop interactions with the substrate polypeptide, nucleotide occupancies, and asymmetric particle distributions. **a**) Top: Cryo-EM densities of the AAA+ motor for all processing-state (PS) conformation, colored by Rpt subunit. Substrate-disengaged seam subunits are at lower resolution likely due to higher mobility and various vertical positions between the bottom and top of the spiral staircases. PS_Rpt3_, PS_Rpt6_, and PS_Rpt2_ show larger density gaps between subunits at the seam, likely because they contain two substrate-disengaged seam subunits with potentially high continuous motions. Bottom: Spiral staircase arrangements of pore-1-loop Tyr residues (Phe for Rpt5) for each processing state. Distances between the substrate backbone and the backbone Cα of the pore-1 loop aromatic residue are indicated for the disengaged seam subunits. Interestingly, some seam subunits reside at the bottom, while others are observed toward the top of the staircase. Positions of the pore-loop residues for these seam subunits are approximate due to the higher mobility and consequently lower resolution. **b**) Cryo-EM densities and atomic models showing the ATP binding sites of all Rpts in the six processing states. Images are focused on the subunit interfaces, with the nucleotide-binding residues of a respective Rpt to the right of the nucleotide and interacting residues of the clockwise-next subunit, including Arg fingers, on the left. The nucleoside portions are shown in green for ADP and red for ATP, the phosphodiesters are depicted in orange and red, and the Mg^2+^ as a green sphere. **c**) Determining the identity of the nucleotide bound to Rpt3 in PS_Rpt5_ and PS_Rpt2_ was more ambiguous due to lower-resolution, but the observed densities fit ADP-Mg^2+^ (left) better than ATP-Mg^2+^ (right). **d**) Particle numbers observed for each processing state in the separate cryo-EM data sets for the 26S proteasome in the presence of sub-stoichiometric TXNL1, 30 s after incubations with the FAT10 substrate (top) or in the presence of excess TXNL1, 2 min after incubation with FAT10 (bottom). **e**) Plotted distances between the substrate backbone and the backbone Cα of the pore-1 loop aromatic residue for Rpt1-6 in each substrate-processing state of the proteasome. Source data

In both datasets, 30 s and 2 min after substrate addition, we observed non-substrate-engaged proteasomes in the RS conformation, with Rpt3 at the top of the staircase, Rpt2 at the bottom and Rpt6 as the seam subunit bound to ADP, while all other subunits were ATP bound. The substrate-engaged proteasomes in both datasets showed six staircase conformations represented by vastly different particle populations (Fig. 5 and Extended Data Fig. 10d). The resolutions in locally refined maps, focusing on the AAA+ motor, were for nonseam subunits generally high enough to reliably determine the identities of the bound nucleotides and distinguish between ADP and ATP (Extended Data Fig. 10b and Tables 1 and 2). Where nucleotide density was weaker, we used the invariant position of the Mg^2+^, which is coordinated by a conserved Thr in both ATP-bound and ADP-bound active sites, to distinguish it from an ATP γ-phosphate. For more mobile seam subunits, nucleotide density was more difficult to determine and we inferred ADP on the basis of the subunit’s staircase position, an open interface to the clockwise-next Rpt and retracted arginine fingers.

**Table 2:** Cryo-EM data collection, refinement and validation statistics and atomic models for the 26S proteasome at 30 s after adding FAT10–Eos substrate and NUB1 cofactor

	PS_Rpt3+_ (AAA+ local)	PS_Rpt6_ (AAA+ local)	PS_Rpt4_ (AAA+ local)	PS_Rpt5_ (AAA+ local)	PS_Rpt1_ (AAA+ local)	PS_Rpt2_ (AAA+ local)
EM Data Bank	EMD-47725	EMD-47723	EMD-71584	EMD-71537	EMD-71538	EMD-47727
PDB	9E8N	9E8K	9PF1	9PDL	9PDN	9E8Q
**Dataset**	26S proteasome with FAT10–Eos substrate and NUB1 cofactor incubated for 30 s					
Magnification	×81,000	×81,000	×81,000	×81,000	×81,000	×81,000
Voltage (kV)	300	300	300	300	300	300
Electron exposure (e^−^ per Å^2^)	50	50	50	50	50	50
Defocus range (μm)	−0.7 to −2.0	−0.7 to −2.0	−0.7 to −2.0	−0.7 to −2.0	−0.7 to −2.0	−0.7 to −2.0
Pixel size (Å)	1.048	1.048	1.048	1.048	1.048	1.048
Symmetry imposed	*C*_1_	*C*_1_	*C*_1_	*C*_1_	*C*_1_	*C*_1_
Initial particles (*n*)	1,159,433	1,159,433	1,159,433	1,159,433	1,159,433	1,159,433
Final particles	15,623	10,722	16,398	66,749	26,687	31,377
Map resolution (Å) FSC threshold	3.62 0.143	4.08 0.143	3.68 0.143	2.76 0.143	3.06 0.143	3.16 0.143
**Refinement**						
Model resolution (Å) FSC threshold	3.4 0.5	3.9 0.5	3.4 0.5	2.7 0.5	3 0.5	3.1 0.5
Map sharpening *B* factor (Å^2^) (determined)	−35.2	−25.6	−34.6	−48.1	−35.6	−43
Initial model used (PDB)	8USC	8USC	8USC	8USC	8USC	8USC
Nonhydrogen atoms	32,822	31,852	32,711	33,269	33,115	80,403
Residues	4,201	4,080	4,184	4,224	4,224	10,175
Ligands	Zn^2+^: 1 Mg^2+^: 2 ATP: 2 ADP: 4 UNK: 14	Zn^2+^: 1 Mg^2+^: 2 ATP: 2 ADP: 4 UNK: 8	Zn^2+^: 1 Mg^2+^: 2 ATP: 2 ADP: 4 UNK: 12	Zn^2+^: 1 Mg^2+^: 5 ATP: 3 ADP: 3 UNK: 11	Zn^2+^: 1 Mg^2+^: 5 ATP: 4 ADP: 2 UNK: 11	Zn^2+^: 1 Mg^2+^: 3 ATP: 2 ADP: 4 UNK: 22
*B* factors (Å^2^) (min/max/mean)						
Protein	64.75/308.37/159.51	30.00/191.54/68.65	30.00/224.88/72.43	43.03/262.47/116.41	36.83/232.66/98.36	4.70/325.03/155.52
Ligand	86.08/201.04/121.76	20.00/276.72/79.74	20.00/183.00/64.68	54.42/266.33/94.96	43.25/130.03/74.14	15.67/202.00/77.12
Root-mean-square deviation						
Bond lengths (Å)	0.004	0.005	0.005	0.005	0.004	0.005
Bond angles (°)	0.9	1.104	1.112	1.047	1.028	1.129
MolProbity	2.66	2.25	2.64	2.43	2.44	2.82
Clashscore	19.66	18.44	17.76	15.21	15.32	25.68
Poor rotamers (%)	3.24	0.06	3.57	2.57	2.91	3.85
Ramachandran plot						
Favored (%)	92.26	92.03	92.53	94.97	94.17	92.35
Allowed (%)	7.07	7.37	7.11	4.67	5.57	7.47
Disallowed (%)	0.68	0.60	0.36	0.36	0.26	0.18

PS_Rpt5_ was clearly the dominant state, with particle numbers ~2 times higher than expected for equal probabilities of states and 4–5 times higher than three of the least populated motor states (Fig. 5 and Extended Data Fig. 10d). If the numbers of particles in a particular conformation correlate with the lifetime of that state during ATP hydrolysis and the spiral-staircase progression in the Rpt hexamer, we postulate that the proteasomal ATPase motor functions by an asymmetric firing mechanism (Supplementary Video 1). This is further supported by varying distributions of nucleotide states, with processing motor conformations containing four ATPs and two ADPs, three ATPs and three ADPs or two ATPs and four ADPs (Fig. 5).

We describe the transition through the observed discrete states in the order matching a presumed counterclockwise progression of ATP hydrolysis, conformational changes and nucleotide exchange events around the Rpt hexamer. Although we describe conformations for an almost complete ATP hydrolysis cycle, we are possibly missing rarer conformations and continuous motion or mobile subunits are difficult to model.

PS_Rpt5_ showed five subunits engaged with the substrate (Rpt5, Rpt1, Rpt2, Rpt6 and Rpt3) and Rpt4 as the disengaged seam subunit (Fig. 5 and Extended Data Fig. 10a). At the top of the staircase, Rpt5, Rpt1 and Rpt2 are ATP bound, whereas Rpt6, Rpt3 and Rpt4 at the bottom have ADP in their active sites. Although the density for Rpt3 is at lower resolution, we can with reasonable confidence assign ADP-Mg^2+^ for its active site (Extended Data Fig. 10b,c). The weak density beyond the β-phosphate does not fit a γ-phosphate, Rpt4’s arginine fingers do not interact with this nucleotide and, most importantly, the counterclockwise-next Rpt6 is convincingly occupied with ADP-Mg^2+^ as well. As there is no solid experimental evidence for nonsequential ATP hydrolysis in an AAA+ motor, an ADP-bound Rpt6 implies that Rpt3 at the bottom of the staircase is also occupied with ADP. Like for the 4D state of the substrate-engaged yeast proteasome^18^, the seam subunit Rpt4 in PS_Rpt5_ is the next subunit to move to the top of the staircase and exchange ADP for ATP. However, the 4D state and the E_D2.1_ state of the human proteasome^20^ both had four ATPs and two ADPs bound to their Rpt subunits, suggesting that, in PS_Rpt5_, an additional ATP hydrolysis event had occurred in Rpt6 without being coupled to movement or nucleotide exchange of Rpt4.

PS_Rpt4_ was not previously observed in structural studies of the substrate-bound yeast or human 26S proteasomes. On the basis of the locally refined map, we could reliably determine that only two subunits, Rpt4 and Rpt5, are ATP bound while the other four Rpt subunits are bound to ADP (Fig. 5). This is a strong deviation from conventional models of always having 4–5 ATP-bound and 1–2 ADP-bound or empty subunits and again suggests that ATP hydrolysis can occur further upward in the spiral staircase and ahead of the large movements and nucleotide exchange of the substrate-disengaged seam subunit. These findings indicate a burst in ATP hydrolysis, whereby additional hydrolysis events occur faster than the movement of seam subunits toward the top of the staircase, possibly because a folded substrate domain at the entrance of the ATPase motor stalls translocation. ADPs, thus, accumulate in up to four subunits, which may subsequently allow several subunits to move from the bottom to the top of the staircase in rapid succession.

The preceding PS_Rpt5_ is the most abundant and likely most long-lived conformation of the hexamer and its transition to PS_Rpt4_ is linked to two ATP hydrolysis events, suggesting that the movement, nucleotide exchange and substrate engagement of Rpt4 are slow steps during substrate processing (Supplementary Video 1). Consistent with this, Rpt4 was previously identified as particularly important for substrate degradation^14^ and our recent single-molecule studies revealed major unfolding defects and increased substrate release when Rpt4’s pore 1 loop was mutated^35^, suggesting that that Rpt4 mediates a power stroke important for mechanical unfolding.

The transition to the subsequent PS_Rpt3*_ state involves Rpt3 moving to the top of the staircase. Because of the limited resolution for Rpt3 (~3.6–4 Å), the identity of the bound nucleotide is ambiguous and the observable density best agrees with ADP (Extended Data Fig. 10b), despite this subunit being at the top of the staircase. It is conceivable that PS_Rpt3*_ reflects a state immediately before nucleotide exchange and substrate reengagement by Rpt3. Although we cannot interpret the side-chain orientations for its pore 1 loop because of limited resolution, the backbone appears positioned close to the substrate and ready to engage (Extended Data Fig. 10a,e). Consequently, two subunits, Rpt6 and Rpt3, are disengaged while the other four contact the substrate through their pore 1 loop. As there are no additional hydrolysis or exchange events, PS_Rpt3*_ represents a hexamer with two ATPs and four ADPs, identical to the nucleotide occupancy of PS_Rpt4_ (Fig. 5). Consistent with this, the substrate polypeptide did not move during the PS_Rpt4_-to-PS_Rpt3*_ transition. There may be a PS_Rpt3_ state with a substrate-engaged, ATP-bound Rpt3 at the top of the staircase that leads to substrate movement in the central channel and precedes the PS_Rpt6_ state but is too short-lived for us to observe.

The following PS_Rpt6_ state also showed two ATPs and four ADPs bound to the ATPase hexamer. During the transition from PS_Rpt3*_, two hydrolysis event occurred, in Rpt5 and Rpt4, while Rpt3 and Rpt6 exchanged ADP for ATP (Fig. 5). Interestingly, there are visible gaps at the ADP-bound subunit interfaces Rpt5–Rpt1, Rpt1–Rpt2 and Rpt2–Rpt6 (Extended Data Fig. 10a). Furthermore, Rpn1 is highly mobile, likely because its anchoring Rpt1 and Rpt2 subunits are at the staircase seam and simultaneously disengaged from the substrate (Extended Data Fig. 10a,e). Because of these higher flexibilities in PS_Rpt6_ and the consequently lower overall resolution for Rpt subunits even in the locally refined map, the nucleotide densities are more ambiguous (Extended Data Fig. 10b), yet our nucleotide assignments are reasonable assumptions on the basis of the observed densities. For instance, in Rpt4, there is no clear density for Mg^2+^, γ-phosphate or the arginine fingers of the neighboring Rpt5 subunit, suggesting that Rpt4 is ADP bound, despite the nucleotide-binding DGF motif apparently being inserted. Either we cannot observe a bound ATP because of limited resolution or PS_Rpt6_ represents a state immediately after ATP hydrolysis in Rpt4.

In PS_Rpt2_, Rpt2 and Rpt6 are ATP bound, while the other four subunits are bound to ADP, meaning that one hydrolysis event and one nucleotide exchange occurred during the transition from PS_Rpt6_. Rpt2, Rpt6, Rpt3 and Rpt4 are in contact with the substrate, whereas Rpt5 and Rpt1 are disengaged (Fig. 5). Interestingly, Rpt1 is rotated up toward the top of the staircase (at level with Rpt2), with its pore 1 loop residue Y249 at ~20 Å from the substrate and potentially sequestered by interaction with Rpt2 (Extended Data Fig. 10a,e). Hence, during the transition from PS_Rpt6_ to PS_Rpt2_, Rpt2 and Rpt1 moved together to the top, possibly because of their coupling by the associated Rpn1 subunit. Rpt5 at the bottom of the staircase is disengaged, yet very close to the substrate (~8 Å; Extended Data Fig. 10a,e). A similar staircase conformation with disengaged Rpt5 and Rpt1, referred to as 1D*, was observed for the yeast proteasome^18^ but assumed to be off pathway. Here, we detected three states, PS_Rpt3*_, PS_Rpt6_ and PS_Rpt2_, with two disengaged subunits and similar staircase states, such as E_D0_ and E_D1_ (ref. ^20^), were previously reported for the human proteasome. Transitions with two disengaged seam subunits may therefore be common or even an important principle for substrate translocation that includes bursts of hydrolysis and Rpt subunit movements.

In PS_Rpt1_, five subunits, Rpt1, Rpt2, Rpt6, Rpt3 and Rpt4, engage the substate and Rpt5 is the seam subunit, albeit at the top of the spiral staircase, ATP bound and ready to engage (Fig. 5 and Extended Data Fig. 10a,e). This state, thus, immediately precedes PS_Rpt5_. There are four subunits, Rpt5, Rpt1, Rpt2 and Rpt6, bound to ATP, while Rpt3 and Rpt4 are ADP bound. PS_Rpt1_ is, therefore, directly comparable to the previously reported human E_D2.0_ (ref. ^20^) and yeast 5T (ref. ^18^) states.

## Discussion

Our studies revealed how the redox-active TXNL1 interacts with the human 26S proteasome in a highly conformation-specific manner, which provides exciting insights into not only the coordination of proteasomal activities and cofactor functions but also the operating principles of the AAA+ ATPase motor. In the RS proteasome, TXNL1’s PITH domain makes transient ionic contacts with Rpn2 and Rpn10, yielding low-affinity binding with at least two orientations above Rpn11 that may allow dynamic association and dissociation. By contrast, in PS proteasomes, the PITH domain gains high affinity through additional interactions of its C-terminal tail with Rpn11’s catalytic groove and a coordination of the active-site Zn^+^. TXNL1 thereby takes advantage of the conformational switch in Rpn11’s Ins-1 region, which controls deubiquitination and inversely coordinates it with TXNL1 binding. In the RS, the open-loop conformation of the Ins-1 region facilitates ubiquitin binding and cleavage but disfavors TXNL1 tail interactions, whereas, in PS proteasomes, the closed Ins-1 region interferes with deubiquitination yet supports TXNL1 tail binding. Interestingly, this holds true for all PSs and spiral-staircase registers of the ATPase motor, except for PS_Rpt2_, in which a slightly larger distance between Rpn11 and the Rpt4/Rpt5 coiled coil may allow an alternative, more relaxed Ins-1 loop conformation that disfavors TXNL1 tail binding and likely accommodates deubiquitination.

**Fig. 6:**
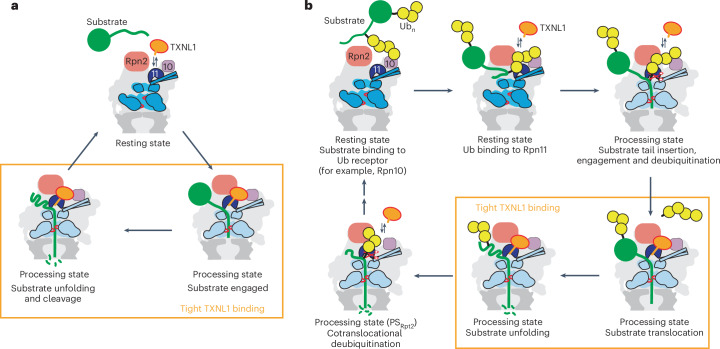
Model for the coordination of proteasome conformational changes, deubiquitination and TXNL1 binding. **a**, TXNL1 loosely interacts with Rpn2, Rpn10 and Rpn11 of the RS proteasome and tightly binds PS proteasomes during substrate unfolding and translocation through additional contacts of its C-terminal tail with Rpn11’s catalytic groove, allowing a potential codegradational reduction of oxidized substrates. **b**, The conformationally selective binding of TXNL1 prevents interference with deubiquitination during the turnover of ubiquitin-tagged substrates. After ubiquitin binding to a receptor and Rpn11 in the RS proteasome, the insertion of the substrate’s flexible initiation region into the ATPase motor induces the conformational switch to PSs and subsequent or simultaneous cleavage of the affinity-conferring ubiquitin chain by Rpn11. TXNL1 then binds with high affinity during substrate unfolding and translocation; however, lower TXNL1 affinity and the exclusion of its C-terminal tail from Rpn11 in the PS_Rpt2_ state would allow a cotranslocational removal of any additional ubiquitin chains.

We determined these TXNL1-bound proteasome structures during ubiquitin-independent degradation of a FAT10 substrate; however, in combination with previous structural and functional studies of ubiquitin-dependent degradation, our results suggest a compelling model for the coordinated binding of TXNL1 that does not interfere with deubiquitination, despite using critical features of Rpn11 (Fig. 6). After substrate recruitment to the RS proteasome, a proximal ubiquitin moiety of the ubiquitin chain appears to bind Rpn11 (refs. ^19,33^). TXNL1 does not interfere with this ubiquitin binding to Rpn11, as its tail does not contact the Ins-1 region in this state. Insertion of the substrate’s flexible initiation region and engagement by the ATPase motor trigger the proteasome conformational switch, the onset of translocation and the immediately subsequent or even simultaneous ubiquitin cleavage^11,33,34^, after which TXNL1 can bind Rpn11 with high affinity. In case a substrate contains additional ubiquitin chains, the proteasome would not have to switch back to the RS for their removal, as the PS_Rpt2_ spiral staircase would allow a cotranslocational deubiquitination while TXNL1 is dissociated or transiently shifted to a low-affinity backward position on Rpn11 (Fig. 6b). Thus, conformationally selective binding allows TXNL1 to be present near the motor entrance during substrate processing yet prevents the interference with initial or cotranslocational deubiquitination events, which is also supported by our ubiquitin-dependent degradation experiments that showed no inhibition by TXNL1.

Although a function in reducing proteasomal cysteines cannot be ruled out, TXNL1 more likely reduces substrates during their unfolding and translocation, given the predicted position of the catalytic TRX domain near the entrance to the proteasomal central channel. Future studies will have to address the exact role of TXNL1 in the proteasome context under normal physiological or certain stress conditions. Severe oxidative stress that may lead to a prevalence of oxidized or crosslinked substrates was reported to cause 26S proteasome dissociation and the isolated 20S CP rather than the 26S proteasome is assumed to fulfill the task of degrading aggregated proteins^36^. Furthermore, TXNL1 levels decrease after arsenic treatment of mammalian cells^31^, suggesting that it has roles under normal physiological conditions rather than being a stress regulator. Indeed, knockdown of *TXNL1* leads to a moderate accumulation of polyubiquitinated substrates^29^. Although the cytosol is an overall reducing environment, the release of oxidizing compounds from peroxisomes, mitochondria or the endoplasmic reticulum may cause local differences in the redox state that require the action of a TRX-like protein for efficient degradation. It was previously shown that the 26S proteasome can process unstructured polypeptides that are linked by two or even three disulfide bonds^37^. However, degradation may be inhibited if disulfides prevent mechanical unfolding by stabilizing individual domains or crosslinking several proteins and TXNL1 may help to resolve these crosslinks in a codegradational manner.

Our structures of the substrate-degrading proteasome also gave important insights into the mechanisms of the ATPase motor, with additional spiral-staircase registers that were absent from previous analyses of stalled motors. All motor states show 2–4 ATP-bound subunits, with the others being ADP bound. Substrate-disengaged seam subunits are never detected nucleotide-free and the ADP-to-ATP exchange, therefore, appears to occur rapidly after a seam subunit moved to the top of the staircase. Transitions between staircase states involve not only single-hydrolysis and nucleotide exchange events but also double-hydrolysis events with one exchange, single hydrolysis without exchange and double exchange without hydrolysis, with either one or two seam subunits moving to the top (Fig. 5). This deviates from the existing hand-over-hand translocation model, which assumes that each translocation step of ~5 Å or 2 aa depends on the coupled occurrence of one hydrolysis event in a subunit near the bottom of the staircase, one seam subunit disengaging from the substrate and moving to the top and one nucleotide exchange by that seam subunit before substrate reengagement^18^. ATP hydrolysis and phosphate release appear to not immediately trigger the seam subunit’s disengagement but instead accumulate strain in the hexamer, especially for the PS_Rpt5_-to-PS_Rpt4_ transition that involves two hydrolysis events. PS_Rpt5_ is ~4–5 times longer lived than the subsequent three states and functional studies implicate Rpt4 (the seam in PS_Rpt5_) in efficient substrate unfolding^14,35^, suggesting that the PS_Rpt5_-to-PS_Rpt4_ transition acts as a power stroke. After rapid passage through PS_Rpt4_, PS_Rpt3*_ and PS_Rpt6_, the AAA+ ring dwells in PS_Rpt2_, where we visualized a defined Eos unfolding intermediate with the N-terminal β-strand extracted from the barrel and a disordered fluorophore environment. Thus, AAA+ ring progression is highly asymmetric, with ~75% of the cycle spent in PS_Rpt2_, PS_Rpt1_ and PS_Rpt5_, involving movement of only Rpt5 and Rpt4 to the top of the staircase (Fig. 5 and Supplementary Video 1). The presence of bursts, with rapid register changes, two consecutive hydrolysis events or tandem seam subunit movements, may thereby facilitate mechanical substrate unfolding. Despite this plasticity, the fundamental translocation step size appears to be ~2 aa, driven by substrate engagement of one seam subunit at a time, which is linked, albeit not always directly, to one ATP hydrolysis event in the ring. On the basis of optical-tweezer studies of the homohexameric ClpX ATPase, we and others previously proposed that these motors can take larger steps^38–42^, yet limitations in spatiotemporal resolution may have prevented the detection of smaller substeps. Indeed, recent nanopore readings of polypeptide translocation by ClpX revealed a 2-aa step size^43^, with overall translocation velocities similar to those observed in previous optical-tweezer experiments.

We propose a stochastic unfolding mechanism in which the 26S proteasome uses fast and slow nucleotide hydrolysis and exchange kinetics, together with pore-loop slipping that prevents seizing on tough substrates^34,35^, for repeated unfolding attempts. This mechanism may yield higher probabilities in overcoming the diverse thermodynamic and kinetic barriers associated with the vast substrate pool of the 26S proteasome. The conformational asymmetries are overall consistent with those observed previously for the DUB-inhibited, substrate-engaged yeast proteasome^18^ or the Usp14-bound human proteasome during ATPγS-inhibited Sic1 substrate degradation^20^, where the PS_Rpt5_-equivalent states 5T and E_D2_ dominated the particle distributions. Remaining differences between datasets are likely because of the type of substrate delivery, the presence of cofactors or distinct substrate-processing stages at the time of proteasome capture. Additional structural landscapes of the 26 proteasomes with more variations in substrates or cofactors will, therefore, be needed to complete the repertoire of motor states, refine our understanding of ATP-hydrolysis-driven degradation and elucidate the sophisticated coordination with enzymatically active cofactors.

## Methods

### Cloning

TXNL1 (Trp32) was gene synthesized with the below amino acid sequence that was codon-optimized for *E*. *coli* expression. TXNL1 DNA was ligated into an expression vector with an N-terminal His–TEV–FLAG (MGSSHHHHHHSSGENLYFQGHMDYKDDDDK), such that a GHMDYKDDDDK overhang was left on the N terminus of TXNL1. All TXNL1 mutants and truncations were generated by site-directed mutagenesis with Q5 polymerase, phosphorylation and ligation.

#### TXNL1 amino acid sequence

MVGVKPVGSDPDFQPELSGAGSRLAVVKFTMRGCGPCLRIAPAFSSMSNKYPQAVFLEVDVHQCQGTAATNNISATPTFLFFRNKVRIDQYQGADAVGLEEKIKQHLENDPGSNEDTDIPKGYMDLMPFINKAGCECLNESDEHGFDNCLRKDTTFLESDCDEQLLITVAFNQPVKLYSMKFQGPDNGQGPKYVKIFINLPRSMDFEEAERSEPTQALELTEDDIKEDGIVPLRYVKFQNVNSVTIFVQSNQGEEETTRISYFTFIGTPVQATNMNDFKRVVGKKGESH

The mEos3.2–titin-tail construct was generated by Gibson assembly of His–TEV mEos3.2 before titin (V15P)-tail–intein–chitin-binding domain (CBD). The titin-tail has one lysine followed by a PPPY motif for Rps5 ubiquitination. The mEos (P141C;C171A)–titin-tail construct was generated by site-directed mutagenesis.

### Purification of TXNL1

TXNL1 was expressed in *E*. *coli* BL21* grown in TB medium by induction with IPTG (250 μM) when cells reached an optical density at 600 nm (OD_600nm_) of 0.6–0.8, followed by shaking (200 rpm) overnight at 16 °C. *E*. *coli* cells (from ~2 L) were resuspended in 30 ml of lysis buffer (60 mM HEPES pH 7.4, 500 mM NaCl, 5% glycerol, 0.5 mM TCEP and 20 mM imidazole) with freshly added benzonase, before sonication and clarification to remove insoluble matter. The supernatant was then passed through Ni-NTA resin (~2 ml) by gravity at room temperature (for ~20 min), before extensive washing to remove contaminants (ten column volumes, sequentially with lysis buffer). TXNL1 was eluted with lysis buffer supplemented with 250 mM imidazole (~10 ml). Protease (His–TEV, 250 μg) was added and TXNL1 was dialyzed into 60 mM HEPES pH 7.4, 200 mM NaCl, 5% glycerol and 0.5 mM TCEP at room temperature for 2 h, followed by overnight dialysis in fresh buffer at 4 °C. Dialyzed TXNL1 was flown over equilibrated Ni-NTA resin and the flow through containing cleaved TXNL1 is collected and concentrated for gel filtration using an SD75 16/600 at 4 °C. Protein is stored at −80 °C in 60 mM HEPES pH 7.4, 150 mM NaCl, 25 mM KCl, 10 mM MgCl_2_ and 0.5 mM TCEP as single-use aliquots (typically ~350 μM). Protein concentration is estimated using 280 nm.

### hs26S proteasome purification

As previously described, human 26S proteasome were purified from HTBH–Rpn11 tagged human HEK293 adapted to suspension culture^25,44^. HEK293 cells were grown at 8% CO_2_, 37 °C and 120 rpm shaking in FreeStyle 293 expression medium with 2% (v/v) FBS. Cells were passaged twice a week at 5 × 10^5^ and newly thawed cells were grown with puromycin. Cell pellets from 4 L of culture were resuspended in 60 mM HEPES pH 7.4, 25 mM NaCl, 25 mM KCl, 5% glycerol, 10 mM MgCl_2_, 5 mM ATP and 0.5 mM TCEP, supplemented with benzonase and EDTA-free proteasome inhibitor tablets. After lysis by a Dounce homogenizer (usually 15 times), lysate was sonicated on ice with low amplitude (20%), clarified for 60 min at 4 °C and flowed over preequilibrated Pierce high-capacity streptavidin agarose (3 ml of resin). For preparing low-salt-treated hs26S proteasomes, resin was sequentially washed with lysis buffer five times, whereas, for high-salt-treated hs26S, resin was washed four times with lysis buffer supplemented with 300 mM NaCl and once with lysis buffer without extra NaCl. Protein was eluted by TEV protease cleavage for 60 min at room temperature and gentle agitation followed by concentration of the elution to ~250 μl. Samples were clarified at 21,000*g* for 15 min before fractionation using an S6 increase 10/300 column in lysis buffer. Fractions containing 26S proteasome were concentrated and concentrations were estimated in a Bradford assay with BSA as a standard and assuming a molecular weight of 2.6 MDa for the proteasome. Typically, 10 μl of ~4 μM aliquots were snap-frozen in liquid N_2_ and stored at −80 °C.

### Purification of Eos–titin-tail substrate

Substrate and mutants were expressed as a His–TEV–Eos–titin I27(V15P)-tail–intein–CBD fusion in *E*. *coli* BL21* by growing cells at 37 °C to OD_600nm_ of 0.6, before cooling cells to 16 °C for ~1 h and induction with IPTG at 0.25 μM. Cells were harvested after 18 h of induction, followed by lysis with sonication (12 cycles of 70% amplitude, 10 s on and 45 s off on ice) in 50 mM HEPES, 300 mM NaCl, 5% glycerol, 10 mM MgCl_2_ and 20 mM imidazole with freshly added EDTA-free proteasome inhibitor tablets and benzonase. Lysate was clarified before flowing over Ni-NTA resin by gravity. Ni-NTA resin was washed extensively with lysis buffer without inhibitor tablets and benzonase. Protein was eluted with lysis buffer supplemented with 250 mM imidazole and bound to chitin resin before washing with five column volumes of lysis buffer. Resin was suspended in lysis buffer overnight with the addition of 50 mM DTT and His–TEV protease. Columns were moved to room temperature for 1 h, before collecting elution and flowing over freshly equilibrated Ni-NTA resin to remove uncleaved protein and His–TEV protease. The cleaned elution was concentrated using Amicon concentrator (30-kDa cutoff) before fractionation using an SD200 16/600 column in gel-filtration buffer (60 mM HEPES pH 7.4, 25 mM NaCl, 25 mM KCl, 5% glycerol, 10 mM MgCl_2_ and 0.5 mM TCEP). After concentrating to ~10 mg ml^−1^, protein was snap-frozen liquid N_2_ as single-use aliquots and stored at −80 °C

### Additional proteins previously established

Fat10, NUB1, Sortase, TEV protease, 3C protease, His–ULP1 protease, Ubiquitin, Rsp5, His–TEV–mE1, Ubch7 and Ub-intein were expressed and purified using previously established protocols^11,25^. Ub-PRG was purified as previously described^25^.

### Western blot

Samples were separated by SDS–PAGE and transferred to PVDF membranes (Thermo Scientific), before blocking in 5% milk TBS-T. Anti-TXNL1 primary antibodies (15289-1-AP, Thermo Scientific) were incubated at room temperature and 1:1,000 dilution with the membranes for 90 min in 5% milk TBS-T, before five washes for 5 min each (~10 ml in TBS-T) and incubation with secondary anti-rabbit horseradish peroxidase (HRP)-conjugated antibodies (ab79773, Abcam) at 1:10,000 dilution for 60 min. After more washes, blots were visualized using HRP after 2 min of incubation.

### Sortase labeling

FAM-labeled peptide (FAM-HHHHHHLPETG) was added N-terminally to TXNL1 and the Eos–titin-tail substrate using Sortase ligation at room temperature for 30 min. Proteins (30–100 μM) were incubated in 50 mM HEPES pH 7.4, 100 mM NaCl, 10 mM CaCl_2_, 0.5 mM TCEP with 500 μM peptide and 25 μM Sortase in 250–450-μl reaction volumes. Labeled proteins were bound to Ni-NTA, washed several times with 60 mM HEPES pH 7.4, 25 mM NaCl, 25 mM KCl, 5% glycerol, 10 mM MgCl_2_ and 20 mM imidazole and eluted with the same buffer supplemented with 250 mM imidazole. Labeled proteins were concentrated and fractionated by gel filtration using an SD75 increase 10/300 in 60 mM HEPES pH 7.4, 25 mM NaCl, 25 mM KCl, 5% glycerol and 10 mM MgCl_2_ to remove excess peptide. Labeled substrates were concentrated, snap-frozen in liquid N_2_ and stored at −80 °C as 10-μl single-use aliquots. For TXNL1 constructs, 0.5 mM TCEP was included throughout the purification and in the storage buffer.

### Insulin reduction assay

When reduced, the B chain of insulin will self-aggregate and precipitate, leading to increased turbidity as measured by absorbance at 650 nm. Insulin (I0516, Sigma Aldrich) at ~1.7 mM was diluted to 30 μM in reaction buffer (60 mM HEPES pH 7.4, 150 mM NaCl and 5% glycerol with 1 mM DTT, unless otherwise stated) with TXNL1 protein. The absorbance at 650 nm was measured every 30 s for at least 2 h at 30 °C.

### Substrate ubiquitination and degradation

Two reactions of His–mE1 (1 μM), Ubch7 (10 μM), Rsp5 (6 μM) and ubiquitin (200 μM) were incubated with Eos–titin-tail substrate (5 μM) for ~30 min at room temperature in ubiquitination reaction buffer (60 mM HEPES pH 7.4, 25 mM NaCl, 25 mM KCl, 10 mM MgCl_2_, 0.5 mM TCEP and 5 mM ATP). Ubiquitinated Eos–titin-tail substrate was mixed with 2× concentrated hs26S proteasome (which was treated before with 2.5 μM Ub-PRG for 30 min) in a prewarmed 384-well plate, and degradation was measured in a BMG Labtech CLARIOstar plate reader at 30 °C by monitoring the emission at 520 nm after excitation with at 500 nm, using the MARS data analysis software. To monitor formation of peptide products by SDS×PAGE analysis, N-terminally FAM-labeled ubiquitinated Eos–titin-tail substrate was used and samples were boiled before gel loading to ensure Eos was fully unfolded and no longer fluorescent.

### Degradation of FAT10 in the presence of arsenite

Sodium (meta)arsenite (S7400, Sigma) was diluted to 4 mM in reaction buffer (60 mM HEPES pH 7.4, 25 mM NaCl, 25 mM KCl, 5% glycerol, 10 mM MgCl_2_, 0.5 mM TCEP and 5 mM ATP). For substrate stocks, FAT10 (10 μM) and NUB1 (10 μM) were mixed and preincubated for at least 15 min on ice before starting the degradation reactions. For the proteasome stock, hs26S proteasome (400 nM) was mixed with TXNL1 (20 μM). To start the degradation reactions, several samples were prepared by mixing the working stocks for NaAsO_2_ (5 μl), 26S proteasome (5 μl) and substrate (10 μl) for a final reaction composition of 1 mM NaAsO_2_, 100 nM 26S proteasome, 5 μM TXNL1, 5 μM FAT10 and 5 μM NUB1 in reaction buffer. Samples were incubated at 30 °C until quenched at indicated time points with 20 μl of SDS–PAGE loading buffer.

### ^FAM^TXNL1 proteasome-binding assay

FAM-labeled TXNL1 and hs26S proteasome were incubated at 2× concentration such that, once diluted 1:1 with buffer or substrate, their final concentrations were at 50 nM for TXNL1 (unless otherwise stated) and 150 or 500 nM for hs26S proteasome. FAT10 substrate was prepared for degradation by incubating the FAT10 with Nub1 at 1:1.2 ratio for at least 15 min at 2× concentration. Reactions were initiated by adding buffer or the preincubated substrate to TXNL1 with or without hs26S proteasome and FP was recorded by measuring the emission at 535 nm after excitation at 480 nm in a preheated (30 °C) 384-well black plate (Costar) using a BMG Labtech CLARIOstar plate reader with MARS data analysis software.

### Ub-TAMRA cleavage by Rpn11

The Rpn11/Rpn8 heterodimer was recombinantly expressed and purified as previously described^16^. Cleavage of Ub-TAMRA (100 μM) by the Rpn11/Rpn8 dimer (0.5 μM) in the absence or presence of TXNL1 (633 μM) or a peptide derived from TXNL1’s C terminus (TNMNDFKRVVGKKGESH, 633 μM) was measured by FP at 30 °C in a 384-well low-volume black flat-bottom plate (Corning, 3820) using a CLARIOstar Plus plate reader (BMG Labtech).

### Cryo-EM sample preparation and data collection

Human 26S proteasome (4 μM, high-salt-washed as described previously) with recombinant FLAG–TXNL1 (16 μM) was diluted 1:1 with preincubated FAT10–Eos (12 μM) and NUB1 (16 μM) for ~ 105 s before plunge-freezing. Sample buffer used was 20 mM HEPES pH 7.4, 25 mM NaCl, 25 mM KCl, 5 mM MgCl_2_, 2 mM ATP, 2.5% glycerol and 0.02% NP-40. Final samples had 2 μM 26S proteasome and 3.5 μl were applied to glow-discharged (glow discharging: 25 mA, 25 s) UltrAufoil R 2/2, 200-mesh, Au grids (Q250AR2A, EM Sciences). Grids were plunge-frozen in liquid ethane (cooled with liquid nitrogen) using a Vitrobot (Thermo Fisher) at 12 °C with 3 s of blot time. Data were collected as previously described^25^; briefly, clipped grids were transferred to a Titan Krios transmission EM instrument operated at 300 keV (Thermo Fisher) equipped with a Gatan K3 and an energy filter (GIF quantum). Images were taken using SerielEM^45^ at a nominal magnification of ×81,000 (1.048 Å pixel size) in super-resolution mode with a defocus ranging from −0.5 to −1.7 μm. We collected 50 frames per shot with a total electron dose ~50 e^−^ per Å^2^ per s. A total of 9,239 videos were collected for the high-salt washed proteasomes with excess TXNL1 and 2 min after addition of the NUB1–FAT10 substrate. The dataset for low-salt-washed proteasomes with substoichiometric amounts of TXNL1 and at 30 s after substrate addition was previously collected^25^.

### Cryo-EM data processing

Data processing was performed using cryoSparc (version 4.4)^46^ and datasets were processed separately. Videos were corrected with patch motion and patch CTF, before blob picking and particle extraction with a box size of 600 (binned by 2×). Particles were sorted by multiple rounds of two-dimensional classification before ab initio 3D reconstruction and heterogenous refinement with ten classes and a refinement box size set to 128. Each class was refined by homogeneous refinement and high-resolution classes of 26S and 30S proteasomes were selected, pooled and extracted with a box size of 600 and no binning. All particles were refined using nonuniform refinement in *C*_2_. Particles were symmetry-expanded in *C*_2_, followed by recentering such that the proteasome RP was in the center of the box, and subsequently extracted with a box size of 340. Particles were then subjected to 3D reconstruction to ensure their correct position and subjected to heterogeneous refinement with ten classes. Classes were pooled on the basis of the global conformation of the RP, either belonging to the RS or PS and refined using nonuniform refinement.

To separate PS proteasomes into individual states, a generous mask for the RP was generated for local refinement and aligned particles were subject to alignment-free 3D classification (ten classes, filtered at 6 Å), from which four dominant classes emerged. A mask for the ATPase domains was then generated for each class, followed by local refinement and another round of 3D classification (ten classes, filtered to 6 Å). Each of the ten classes was refined with local refinement and similar states (no obvious differences) were combined, such that six ATPase motor conformations were achieved. ATPase 3D classification for PS_Rpt5_ resulted in different classes with varying positions of the seam subunit Rpt4 and the PS_Rpt1_. For PS_Rpt6_, multiple poor-quality particles were included likely because of lower resolution. Therefore, another round of heterogenous refinement and local refinement of the ATPase was performed and resulted in a relatively high-resolution PS_Rpt6_ conformation, while the remaining particles appeared to be junk or low-resolution data. For each processing conformation, an RP mask was generated and local refinement was used to generate final unsharpened maps. An ATPase motor mask was used for processing maps from the 30S dataset^25^. While we describe distinct states of the 26S proteasome, it is important to note that alignments of particles and 3D reconstructions can be dominated by more rigid parts of a protein complex. In addition, within each discrete state, there is continuous motion present throughout the 26S proteasome complex and this can vary depending on the defined conformational state described. For example, Rpn10 and Rpn1 can be at lower resolutions relative the AAA+ motor. It should be noted that because of current classification techniques, particles are sorted on the basis of their probability of belonging to each assigned state and more discrete states might be observed when increasing particle numbers. Although several groups previously generated composite maps from individually locally refined maps^18,20^, we did not use this approach. However, for guidance in model building and map representation and to aid with the anisotropy in map resolution, we used maps sharpened with deepEMhancer^47^ but always validated model interpretations with unprocessed maps and locally refined maps.

For the 30S dataset with partial occupancy of TXNL1 on Rpn11, we also generated masks that encompassed TXNL1, Rpn2, Rpn11 and Rpn10 and used local refinement to improve resolution. Using these locally refined maps, alignment-free 3D classification was used followed by local refinement of each separate class on the basis of the presence or absence of TXNL1. Particles were pooled into two groups, with and without TXNL1, and subjected to local refinement using the same mask for TXNL1. The particle numbers for the presence and absence of TXNL1 were used to roughly calculate the ratio of TXNL1 binding to a particular PS conformation. However, it should be noted that numbers are estimates because of lower resolutions in certain classes and particles being sorted on the basis of the probability of belonging to a certain class.

For the RS proteasome, RP-aligned particles were subjected to alignment-free 3D classification, from which four major classes emerged. Each class was refined by nonuniform refinement, whereby class 1 was considered junk because of broken RP, class 2 was characterized by a flexible Rpn1 and classes 3 and 4 were charactered as high-RS conformations with a shift in the position of the RP when compared. Class 2 was further processed by aligned local refinement with a mask encompassing Rpn10, Rpn2, Rpn11 and the density in between. Aligned particles were subject to alignment-free 3D classification, which generated ten classes of varying resolutions. We chose three classes with interesting features to further refine. Two classes showed the PITH domain of TXNL1 in forward and backward conformations and were refined through local refinement with a whole RP mask. One conformation appeared similar to so-called ‘deubiquitination states’ E_A2_ and E_B_ (ref. ^19^) and showed density near Rpn11, which is likely a ubiquitin-like domain. After local refinement with a whole RP mask, we low-pass-filtered the whole RP refined map to 6 Å and docked the atomic model for the E_B_ state (Protein Data Bank (PDB) 6MSE) using ChimeraX^48^. Local refinement with a mask encompassing Rpn11, the extra density, the coiled coil of Rpt4/5, Rpn10 and Rpn2 and subsequent 3D classification did not further improve the resolution. For classes 3 and 4 with a slight shift in the RP, there was an ensemble of density where we would expect the PITH domain of TXNL1. We kept classes 3 and 4 separate and used local refinement to align particles using a mask encompassing Rpn2, Rpn10, Rpn11 and the ensemble of density in between those subunits. cryoSparc 3D variability and cluster analysis^49^ was used to separate out particles on the basis of the position of the PITH domain. We could characterize two major conformations, a forward and backward conformation. Those states, named RS.1 TXNL1 state 1, RS.1 TXNL1 state 2, RS.2 TXNL1 state 1 and RS.2 TXNL1 state 2, were refined by local refinement with a whole RP mask and maps sharpened with deepEMhancer^47^ along with unprocessed maps were used for representing density.

### Cryo-EM model building and model visualization

Previously built atomic models for human 26S proteasomes^25^ were used as starting models for each proteasome structure. Atomic models were generated for RS proteasome maps RS.1 TXNL1 state 1, RS.1 TXNL1 state 2, RS.2 TXNL1 state 1 and RS.2 TXNL1 state 2. Briefly, models were fit using ChimeraX Fit to map and individual subunits were manually adjusted using the same function. TXNL1’s PITH domain was truncated from a model generated by AlphaFold^50^. Then, phenix.refine^51^ with simulated annealing and morphing turned on for the first round was used to adjust models, followed by several rounds of iterative model building in Coot^52^ and real-space refinement in PHENIX without simulated annealing and morphing. For atomic models of the PS proteasomes, deepEMhancer maps were used to guide model building. For AAA+ domains, Rpts were separated into multiple parts and rigid bodies using ChimeraX Fit to map, followed by several rounds of manual adjustment in Coot. Once roughly assembled and fit, models were subjected to multiple rounds of manual adjustment in Coot and real-space refinement with PHENIX (refinement was typically conducted using unprocessed maps). PredictAndBuild in PHENIX was used to model Eos with AlphaFold-generated models before manual adjustment with Coot. The chromophore for Eos (code CR8 in Coot) was fit and adjusted in Coot, followed by PHENIX real-space refinement using a restraints cif file generated in Phenix. PyMol (version 1.8, Schrödinger), UCSF chimera^53^ and ChimeraX^48^ were used to generate figures. Local resolutions were calculated using cryoSparc local resolution estimation with a Fourier shell correlation (FSC) criterion of 0.143 and displayed on locally filtered maps (using a *B* factor obtained during refinement) in ChimeraX with a colored surface.

### Statistics and reproducibility

SDS–PAGE gels and western blots shown in Extended Data Figs. 1e and 5a are representatives of at least two technical replicates for. Micrographs from cryo-EM are representative images. Other quantitative measurements presented in Fig. 3 and Extended Data Figs. 1 and 5 are representatives of at least three technical replicates. For Extended Data Fig. 8, the curves are representatives of two technical replicates.

### Reporting summary

Further information on research design is available in the Nature Portfolio Reporting Summary linked to this article.

## Online content

Any methods, additional references, Nature Portfolio reporting summaries, source data, extended data, supplementary information, acknowledgements, peer review information; details of author contributions and competing interests; and statements of data and code availability are available at 10.1038/s41594-025-01695-2.

## Supplementary information

Reporting Summary

Supplementary Video 1 **Supplemental Video 1 (related to Figure 5):** Shown is the morph between the six main conformational states of the proteasomal AAA+ ATPase motor (PS_Rpt2_ → PS_Rpt1_ → PS_Rpt5_ → PS_Rpt4_ → PS_Rpt3_ → PS_Rpt6_) during ATP-dependent substrate degradation, with the timing of each transition based on the observed cryo-EM particle distribution to reflect the probable temporally asymmetric firing of subunits.

## Source data

Source Data Fig. 3 Raw measurement data.

Source Data Extended Data Fig. 1 Uncropped gels and western blots.

Source Data Extended Data Fig. 1 Raw measurement data.

Source Data Extended Data Fig. 5 Uncropped gel.

Source Data Extended Data Fig. 5 Raw measurement data.

Source Data Extended Data Fig. 7 Raw measurement data.

Source Data Extended Data Fig. 8 Raw measurement data.

Source Data Extended Data Fig. 9 Raw measurement data.

Source Data Extended Data Fig. 10 Raw measurement data.

## Data Availability

All data generated or analyzed during this study are included in this manuscript and the Supplementary Information. The cryo-EM density maps and corresponding atomic coordinates for human 26S proteasomes in the presence of excess TXNL1 and actively degrading FAT10–Eos were deposited to the EM Data Bank and PDB under the following accession codes: EMD-47719 and PDB 9E8G (PS_Rpt5_ + TXNL1), EMD-47724 and PDB 9E8L (PS_Rpt4_ + TXNL1), EMD-71581 (PS_Rpt3*_ + TXNL1), EMD-71583 (PS_Rpt6_ + TXNL1), EMD-47726 and PDB 9E8O (PS_Rpt2_ + TXNL1 with Eos substrate resolved), EMD-47722 and PDB 9E8J (PS_Rpt1_ + TXNL1), EMD-47721 and PDB 9E8I (RS + TXNL1 forward), EMD-47720 and PDB 9E8H (RS + TXNL1 backward) and AAA+ aligned map EMD-71534 and PDB 9PDI (PS_Rpt2_ + TXNL1 with Eos substrate resolved). The AAA+ aligned maps and atomic coordinates from the 30S degradation dataset can be found under the following accession codes: EMD-71537 and PDB 9PDL (PS_Rpt5_), EMD-71584 and PDB 9PF1 (PS_Rpt4_), EMD-47725 and PDB 9E8N (PS_Rpt3*_), EMD-47723 and PDB 9E8K (PS_Rpt6_), EMD-71538 and PDB 9PDN (PS_Rpt1_) and EMD-47727 and PDB 9E8Q (PS_Rpt2_). These datasets for the proteasome after 30S degradation contained copurified TXNL1 in substoichiometric amounts. All constructs generated in this study are available from the lead contact upon request and completion of a materials transfer agreement. Source data are provided with this paper.
