# Supplementary figures and images for: Structural landscape of the degrading 26S proteasome reveals conformation-specific binding of TXNL1
Source: Nat Struct Mol Biol. 2025 Nov 6;32(12):2403–15. doi: 10.1038/s41594-025-01695-2 (PMC12700817; doi:10.1038/s41594-025-01695-2)

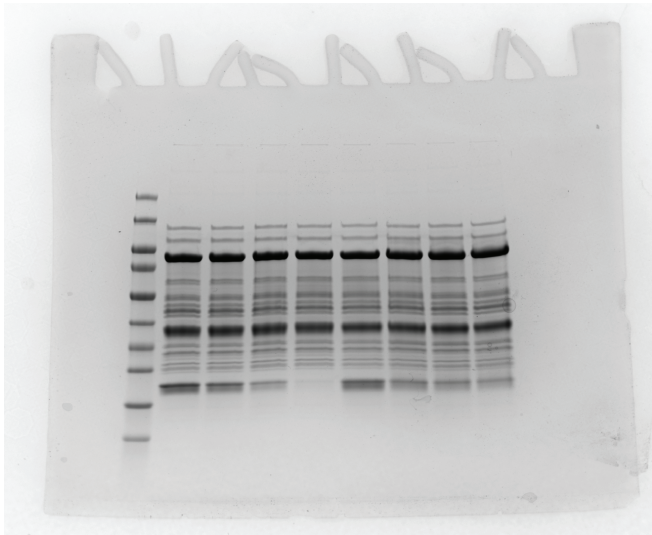

Supplement: Supplementary file 6 — Uncropped gel. [file 41594_2025_1695_MOESM6_ESM.pdf]
